# The identification and neurochemical characterization of central neurons that target parasympathetic preganglionic neurons involved in the regulation of choroidal blood flow in the rat eye using pseudorabies virus, immunolabeling and conventional pathway tracing methods

**DOI:** 10.3389/fnana.2015.00065

**Published:** 2015-06-02

**Authors:** Chunyan Li, Malinda E. C. Fitzgerald, Nobel Del Mar, Sherry Cuthbertson-Coates, Mark S. LeDoux, Suzhen Gong, James P. Ryan, Anton Reiner

**Affiliations:** ^1^Department of Anatomy and Neurobiology, University of Tennessee Health Science CenterMemphis, TN, USA; ^2^Department of Biology, Christian Brothers UniversityMemphis, TN, USA; ^3^Department of Ophthalmology, University of Tennessee Health Science CenterMemphis, TN, USA; ^4^Department of Neurology, University of Tennessee Health Science CenterMemphis, TN, USA; ^5^Department of Microbiology, Immunology and Biochemistry, University of Tennessee Health Science CenterMemphis, TN, USA

**Keywords:** choroidal blood flow, superior salivatory nucleus, pterygopalatine ganglion, pseudorabies virus, parasympathetic, nucleus of solitary tract

## Abstract

The choroidal blood vessels of the eye provide the main vascular support to the outer retina. These blood vessels are under parasympathetic vasodilatory control via input from the pterygopalatine ganglion (PPG), which in turn receives its preganglionic input from the superior salivatory nucleus (SSN) of the hindbrain. The present study characterized the central neurons projecting to the SSN neurons innervating choroidal PPG neurons, using pathway tracing and immunolabeling. In the initial set of studies, minute injections of the Bartha strain of the retrograde transneuronal tracer pseudorabies virus (PRV) were made into choroid in rats in which the superior cervical ganglia had been excised (to prevent labeling of sympathetic circuitry). Diverse neuronal populations beyond the choroidal part of ipsilateral SSN showed transneuronal labeling, which notably included the parvocellular part of the paraventricular nucleus of the hypothalamus (PVN), the periaqueductal gray, the raphe magnus (RaM), the B3 region of the pons, A5, the nucleus of the solitary tract (NTS), the rostral ventrolateral medulla (RVLM), and the intermediate reticular nucleus of the medulla. The PRV+ neurons were located in the parts of these cell groups that are responsive to systemic blood pressure signals and involved in systemic blood pressure regulation by the sympathetic nervous system. In a second set of studies using PRV labeling, conventional pathway tracing, and immunolabeling, we found that PVN neurons projecting to SSN tended to be oxytocinergic and glutamatergic, RaM neurons projecting to SSN were serotonergic, and NTS neurons projecting to SSN were glutamatergic. Our results suggest that blood pressure and volume signals that drive sympathetic constriction of the systemic vasculature may also drive parasympathetic vasodilation of the choroidal vasculature, and may thereby contribute to choroidal baroregulation during low blood pressure.

## Introduction

The choroidal blood vessels in the eye and orbital blood vessels supplying the choroid are innervated by parasympathetic, sympathetic and sensory nerve fibers that adaptively regulate choroidal blood flow (Kirby et al., 1978; Guglielmone and Cantino, 1982; Bill, 1984, 1985, 1991; Stone et al., 1987; Fitzgerald et al., 1990a,b, 1996; Cuthbertson et al., 1996, 1997). Such adaptive control appears to be important for maintaining the health of retinal photoreceptors and maintaining normal visual functioning (Potts, 1966; Reiner et al., 1983; Fitzgerald et al., 1990b; Shih et al., 1993, 1994; Hodos et al., 1998). Numerous studies have shown that the pterygopalatine ganglion (PPG) is the major source of parasympathetic input to the choroid and to periocular vessels in mammals (Ruskell, 1971b; Uddman et al., 1980a; Bill, 1984, 1985, 1991; Stone, 1986; Stone et al., 1987). This parasympathetic input mediates vasodilation by release of vasoactive intestinal polypeptide (VIP) and nitric oxide (NO) (Uddman et al., 1980a; Stone et al., 1987; Yamamoto et al., 1993; Alm et al., 1995).

The PPG receives its preganglionic input from the superior salivatory nucleus (SSN) of the hindbrain via the greater petrosal branch of the facial nerve (Contreras et al., 1980; Nicholson and Severin, 1981; Spencer et al., 1990; Ng et al., 1994; Tóth et al., 1999). The SSN itself is located dorsolateral to the facial motor nucleus. The SSN neurons, which are adjacent to noradrenergic neurons of the A5 cell group, are cholinergic, and a subset have been reported to contain neuronal nitric oxide synthase (nNOS) as well (Gai and Blessing, 1996; Zhu et al., 1996, 1997; Cuthbertson et al., 2003). The PPG, in addition to its innervation of ocular and choroidal blood vessels, also innervates orbital blood vessels, the Meibomian glands, the lacrimal gland, the Harderian gland, blood vessels of the nasal mucosa and palate, and cerebral blood vessels (Ruskell, 1965, 1971a; Uddman et al., 1980b; Ten Tusscher et al., 1990; Nakai et al., 1993; Van Der Werf et al., 1996; Ledoux et al., 2001). Using transneuronal retrograde labeling from the choroid with the Bartha strain of pseudorabies virus (PRV), we have found that the neurons controlling choroidal blood flow via the PPG lie within rostromedial SSN and are largely co-incident with the nNOS+ population within SSN (Cuthbertson et al., 2003).

As part of an effort to elucidate central control of choroidal blood flow via the SSN, we subsequently used the higher order labeling after intrachoroidal PRV, together with complementary conventional pathway tracing methods, to show that the paraventricular nucleus (PVN) of the hypothalamus and the nucleus of the solitary tract (NTS) of the medulla project directly to the prechoroidal neurons of SSN (Li et al., 2010). To better understand the influence of PVN and NTS on choroidal blood flow, in the present study, we characterized the localization within PVN and NTS of the neurons projecting to prechoroidal SSN, as well as their neurotransmitter features. We also used higher-order PRV labeling to identify additional central sites having input to the prechoroidal neurons of SSN. We found that the various higher-order cell groups controlling choroid via the SSN include neuronal populations known to be involved in the sympathetic control of systemic blood pressure and blood flow. Our overall results thus indicate that parasympathetic regulation of choroidal blood flow in the eye by the SSN-PPG circuit is likely to be responsive to the same blood pressure and volume signals that drive sympathetic control of the systemic vasculature. By means of these inputs, the SSN-PPG circuit is likely to contribute to the demonstrated adaptive regulation of ChBF in response to drops in systemic blood pressure or volume (Kiel and Shepherd, 1992; Reiner et al., 2003, 2010, 2011).

## Materials and methods

### Subjects and approach

To identify central parasympathetic neurons involved in the control of choroidal blood flow, a retrograde transneuronal tracer, the Bartha strain of pseudorabies virus (PRV) was used in 6–9 month old adult male Sprague-Dawley rats (300–400 g) from Harlan (Indianapolis, IN). Our goal was to make PRV injections confined to choroid (i.e., with no or only negligible spill outside the choroid), so that the higher order parasympathetic circuitry specifically controlling choroid could be identified. As the choroidal layer of the eye in rats is extremely thin (about 120 μm) (Cheng et al., 2006), restricting injections to the choroid without penetration into the vitreous or reflux into the periorbital space is difficult. As a strategy for achieving our goal of restricted injections into choroid, we varied the amount injected, the gauge of the syringe needle, and the survival time. We targeted choroid in 40 rats in which we also completely removed both superior cervical ganglia, to prevent transport of virus via the sympathetic innervation of the choroid, as has been shown to be effective by others (Tóth et al., 1999; Ledoux et al., 2001; Rezek et al., 2008). To judge whether injections were restricted to choroid, we relied on the published evidence that the spread of choroid to the vitreous, the extraocular muscles or the periorbital facial musculature yields characteristic labeling of motor neuron pools and preganglionic neuron pools in the brain (Graf et al., 2002; Pickard et al., 2002; Billig and Balaban, 2004, 2005; Gonzalez-Joekes and Schreurs, 2012). Additionally, we performed 5 control injections into the vitreous or into the periorbital space (RF21, RF59, RF60, RF69, R10), to help judge extrachoroidal spread of PRV in the 40 cases in which we targeted choroid. We thereby identified 8 rats with PRV injections into choroid with no spread or only negligible spread outside of choroid, and higher order labeling in brain beyond SSN. One of these (RF7) had been used for analysis of SSN labeling in Cuthbertson et al. (2003), and two additional ones (R11, R12) had been used to report on labeling in PVN and NTS in Li et al. (2010). In all 8 cases, novel information is reported here on the neuronal populations containing higher-order labeling in brain after intrachoroidal injection with PRV. All animal studies were performed in accordance with a protocol approved by the Institutional Animal Care and Use Committee of the University of Tennessee Health Science Center, and complied with the National Institutes of Health, Society for Neuroscience guidelines, and the ARVO statement on the Use of Animals in Ophthalmic and Vision Research.

### Pseudorabies virus injection

Rats were anesthetized with an intraperitoneal injection of 0.1 ml/100 g of a ketamine/xylazine mixture (87/13 mg/kg), and the right superior-temporal choroid was injected with 0.2–4.0 μl of PRV (3 × 10^8^ plaque forming units/ml), as described previously (Cuthbertson et al., 2003). In brief, the needle tip was inserted through the conjunctiva and sclera posterior to the ciliary complex into the choroid under visual guidance using a surgical microscope or a magnifying viewer. The tracer was then slowly injected over 3–5 min, and the needle was withdrawn. During injection and withdrawal, the puncture site was monitored for efflux. Any efflux was blotted with a sterile cotton swab, the conjunctival sac was rinsed with sterile normal saline, and the puncture sealed with superglue. In general, the larger injection amounts were used for shorter survivals and the smaller for longer survival, to favor specificity of labeling. Animals were allowed to survive between 52 and 144 h after virus injection. All PRV injections were performed with a Hamilton syringe with a 30- or 32-gauge needle, with the thinner needle proving more reliable for confining injections to the choroid. Two to three weeks prior to PRV injection, the superior cervical ganglia (SCGs) were removed bilaterally, as follows. A single ventral midline neck incision was made to allow access to both the right and left SCG, which lie immediately superior to the bifurcation of the common carotid artery in the upper neck. The cervical portion of the sympathetic trunk and SCG was dissected free from the carotid artery and excised *in toto* on both sides. We have confirmed the efficacy of our SCG removals by showing that they eliminate all sympathetic innervation from the choroid, as detected by immunolabeling for VMAT2, a marker of sympathetic nerve terminals (Hou and Dahlstrom, 1996; Headley et al., 2007). Since PRV does not typically show transganglionic transport via sensory ganglia (Jansen et al., 1992; Rotto-Percelay et al., 1992; Ledoux et al., 2001), we did not transect the ophthalmic nerves.

### Histological tissue preparation

Rats that had received a PRV injection were anesthetized with an intraperitoneal injection of 0.1 ml/100 g of a ketamine/xylazine mixture (87/13 mg/kg). The chest was opened, 0.2 ml of heparinized saline (2400 U.S.P. units/ml) was injected into the heart, and the rat was then transcardially perfused with 0.9% saline followed by 400–500 ml of 4% paraformaldehyde prepared in 0.1 M sodium phosphate buffer (PB) with 0.1 M lysine and 0.01 M sodium periodate (PLP fixative), pH 7.2–7.4. Brains were cryoprotected at 4°C for at least 24 h in a 20% sucrose/10% glycerol/0.138% sodium azide in 0.1 M PB solution. They were subsequently frozen with dry ice and sectioned on a sliding microtome at 40 μm. Sections were collected as 6–12 parallel series, and one series was typically mounted immediately during sectioning on gelatin-coated slides, allowed to dry, and then stained with cresyl violet. The remaining free-floating sections were stored at 4°C in a 0.02% sodium azide/0.02% imidazole in 0.1 M PB solution until they were immunolabeled for PRV.

### Peroxidase-antiperoxidase immunohistochemistry

**Figure 1:**
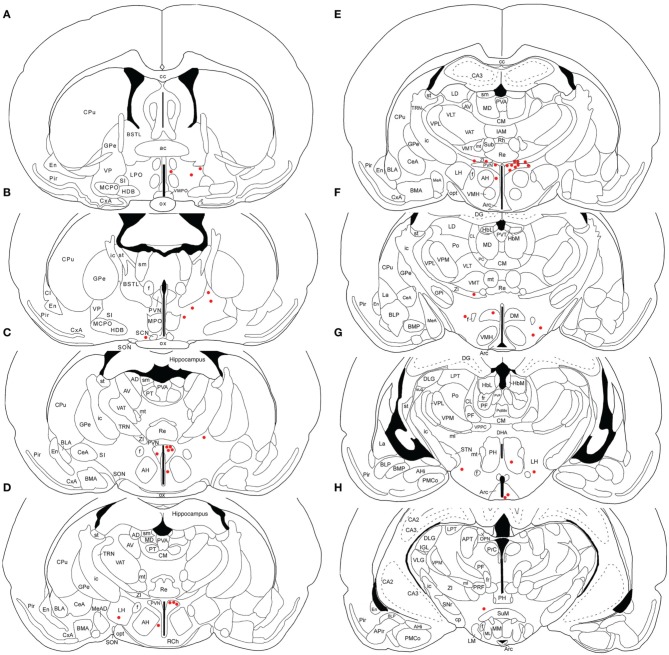
**Line drawing schematics of coronal sections through the rostral and mid telencephalon and diencephalon, based on the rat atlas of Paxinos and Watson (2009), schematizing the distribution of PRV+ neurons in RF73**. Each filled red circle represents one PRV + neuron. The images progress from most rostral at the top left **(A)** to most caudal at the lower right **(H)**. For full structure names see list of abbreviations.

**Figure 2:**
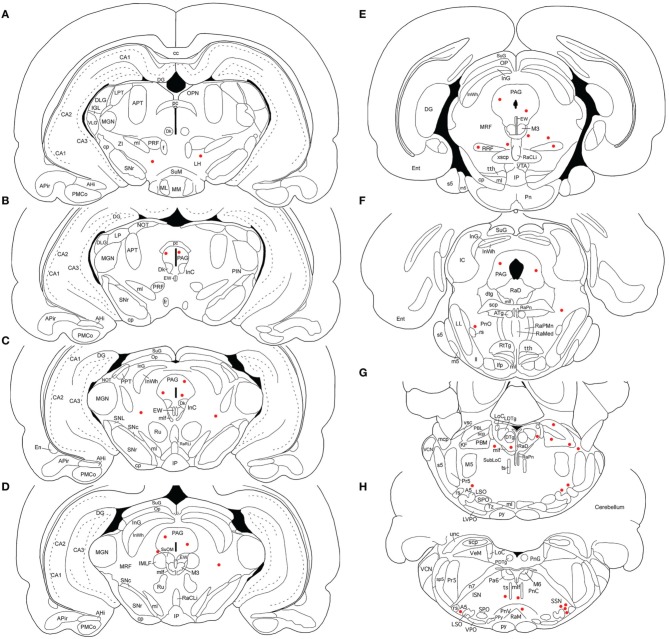
**Line drawing schematics of coronal sections through the midbrain and rostral hindbrain, based on the rat atlas of Paxinos and Watson (2009), showing the distribution of PRV+ neurons in RF73**. Each filled red circle represents one PRV + neuron. The images progress from most rostral at the top left **(A)** to most caudal at the lower right **(H)**. For full structure names see list of abbreviations.

**Figure 3:**
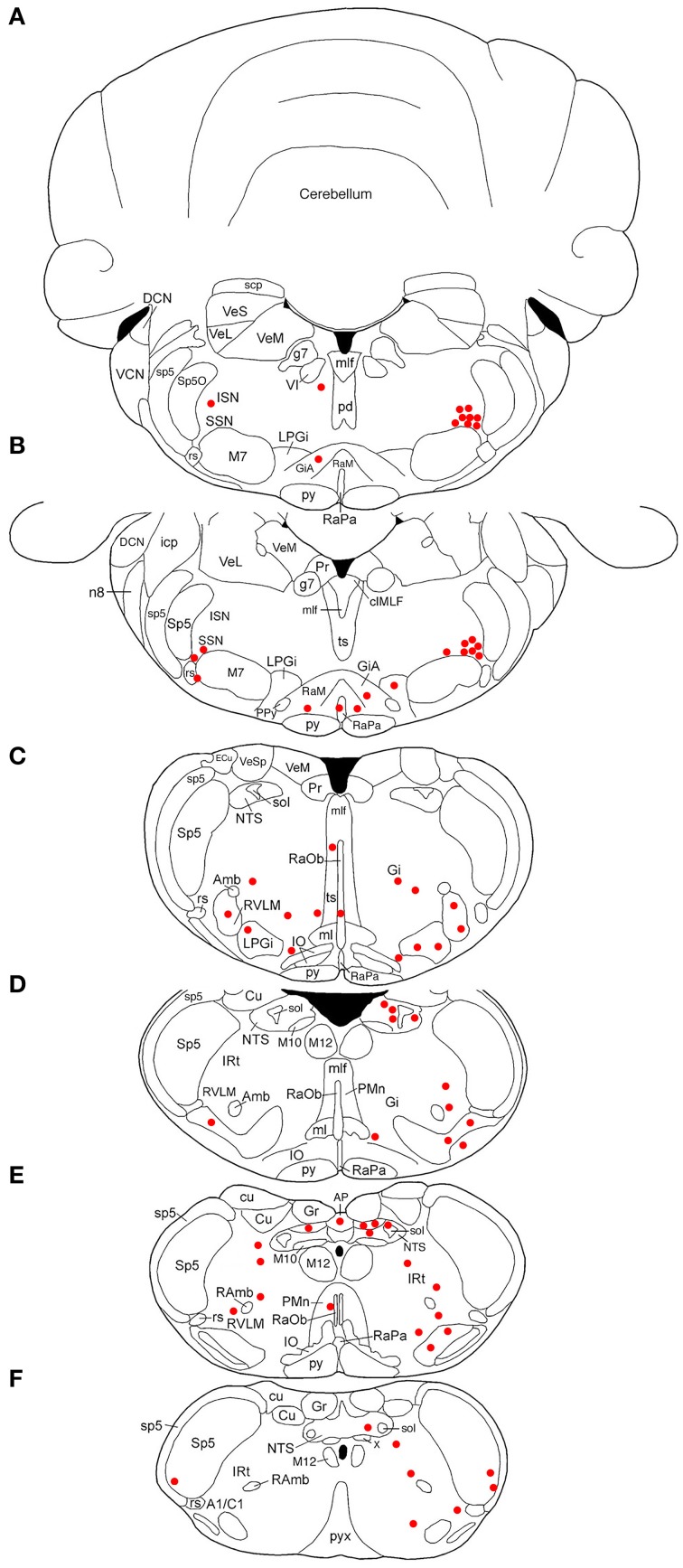
**Line drawing schematics of coronal sections through the caudal pons and medulla, based on the rat atlas of Paxinos and Watson (2009), showing the distribution of PRV+ neurons in RF73, in which the PRV injection was well confined to the choroid and yielded extensive higher-order labeling**. Each filled red circle represents one PRV + neuron. The images progress from most rostral at the top **(A)** to most caudal at the bottom **(F)**. For full structure names see list of abbreviations.

**Figure 4:**
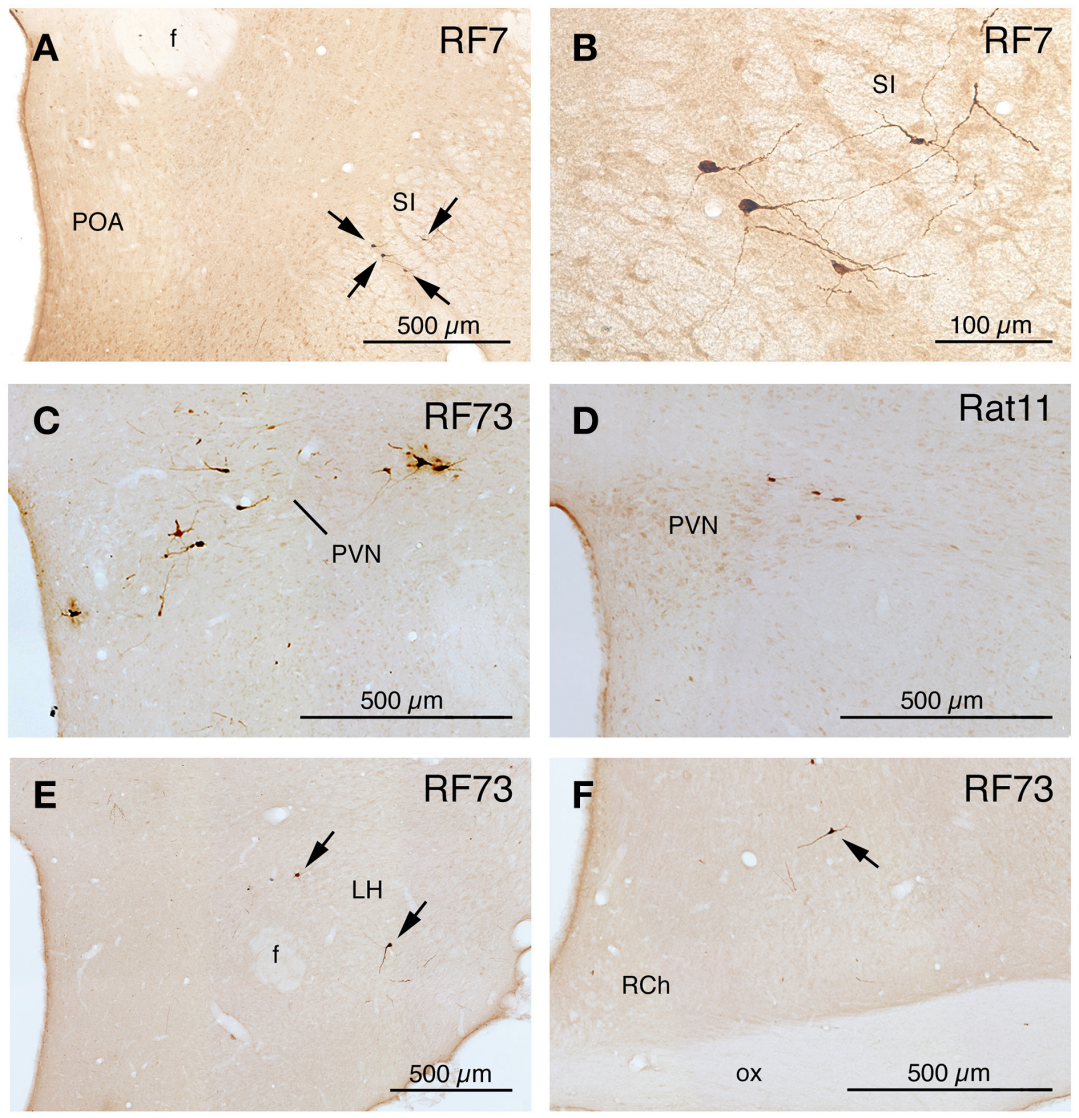
**Images of PRV-labeled neurons in the diencephalon and telencephalon after an intrachoroidal injection of virus into the right eye, with the labeled neurons detected by PAP immunolabeling with DAB**. **(A,B)** Show low and high power views of PRV+ neurons (arrows) in the substantia innominata (SI) of RF7, near the preoptic area (POA). **(C,D)** Show PRV+ neurons in the right paraventricular nucleus (PVN), from RF73 and R11, respectively. **(E,F)** Show PRV+ neurons (arrows) in the lateral hypothalamus (LH) and retrochiasmatic area of the hypothalamus (RCh), from RF73. All images are of coronal sections. Abbreviation: ox, optic chiasm.

**Figure 5:**
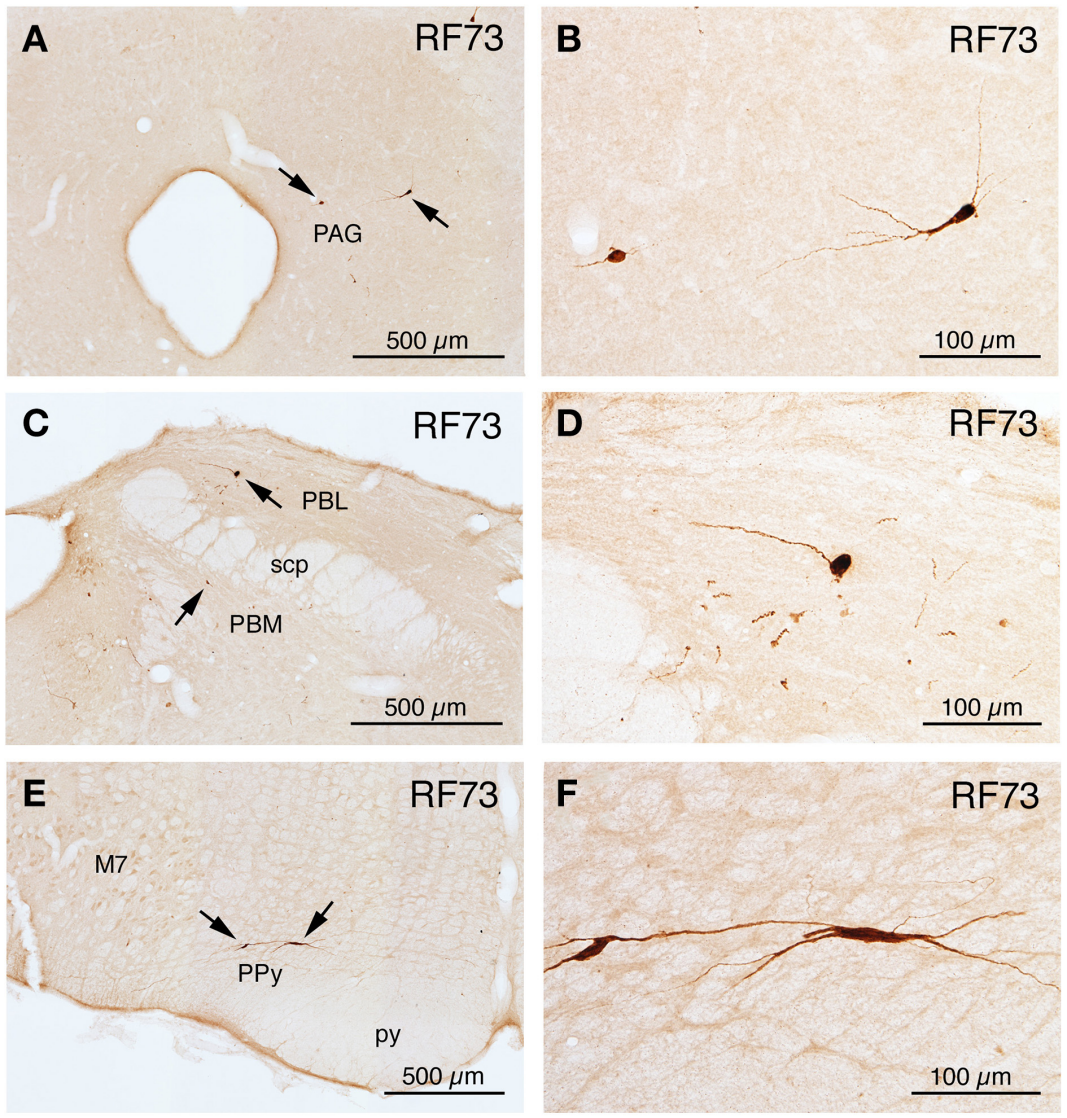
**Images of PRV-labeled neurons at rostral pons and midbrain after an intrachoroidal injection of virus into the right eye, with the labeled neurons detected by PAP immunolabeling with DAB**. **(A,B)** Show low and high power views of PRV+ neurons in the periaqueductal gray (PAG) at a rostral midbrain level. **(C,D)** Show low and high power views of PRV+ neurons (arrows) in the lateral parabrachial nucleus (PBL) lateral to the superior cerebellar peduncle (scp), on the right side of the brain. **(E)** Presents a view of PRV+ neurons in the left parapyramidal region (PPy) of the pons, and image **(F)** presents a higher power view of those PRV+ neurons. All images are from RF73, which had a long survival following a minute PRV injection restricted to choroid. All images are of coronal sections.

**Figure 6:**
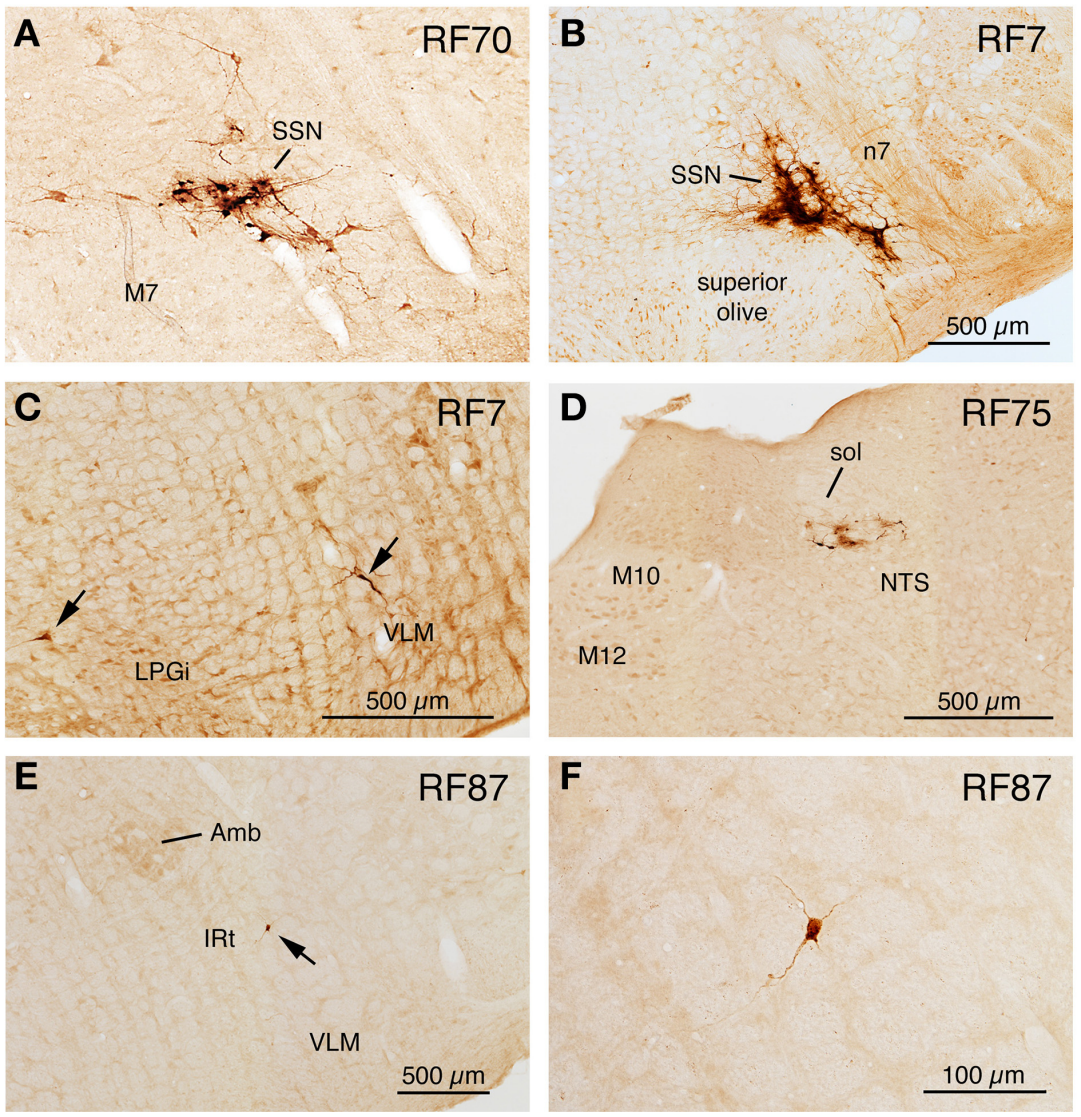
**Images of PRV-labeled neurons in the medulla and caudal pons after an intrachoroidal injection of virus into the right eye, with the labeled neurons detected by PAP immunolabeling with DAB**. **(A,B)** Show low-power views of PRV+ neurons in the right superior salivatory nucleus (SSN) of RF70 (74 h survival) and RF7 (52 h survival). **(C)** Shows PRV+ neurons in the right rostral ventrolateral medulla (RVLM) in RF7. **(D)** Shows PRV+ neurons in the right NTS of RF75 (142.4 h survival after minute intrachoroidal PRV injection). **(E,F)** Show low and high power views of a labeled neuron in the right intermediate reticular nucleus (IRt) of RF87 (73.5 h survival). Arrows indicate PRV+ neurons **(C,E)**. The scale bar in **(B)** provides the magnification for **(A,B)**. All images are of coronal sections.

**Figure 7:**
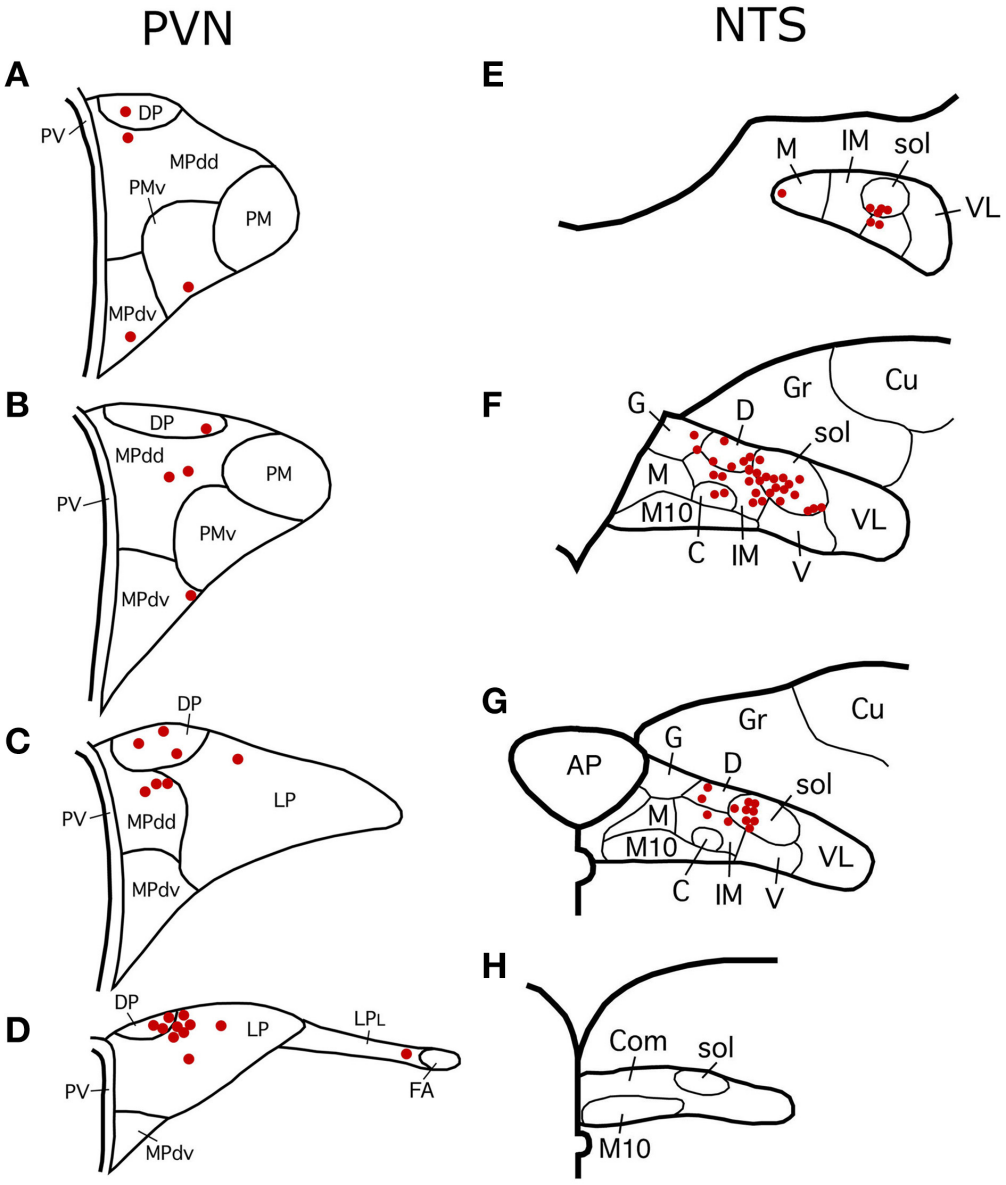
**(A–D)** Schematic distribution of PRV+ neurons in PVN, representing a composite of one series of sections from RF73, and one from RF87. Note that the majority of PRV+ neurons are localized in the parvocellular subdivisions of PVN. Schematic subdivisions of PVN based on Hallbeck et al. (2001). Levels shown in **(A–C)** correspond to the levels shown in Figures 1C–E. **(E–H)** Schematic distribution of PRV+ neurons in NTS. The mapping represents a composite of two series of sections from RF73, a long survival case following a minute PRV injection restricted to choroid. Note that the majority of PRV+ neurons are localized to the dorsal, intermediate and solitary tract subdivisions of NTS. Schematic subdivisions of NTS based on Zhang and Ashwell (2001). Levels shown in **(E–G)** correspond to the levels shown in Figures 3C–E.

Immunohistochemical single-labeling to detect PRV at the light microscopic level was carried out as described previously (Reiner et al., 1991; Cuthbertson et al., 1996). The primary antibody was a highly sensitive and specific goat anti-PRV diluted at 1:15,000–1:100,000 (Ledoux et al., 2001). Preparation and characterization of this antibody have been described previously (Ledoux et al., 2001; Cuthbertson et al., 2003). The diluent was a solution of 0.1 M phosphate buffer/0.3% Triton X-100/0.001% sodium azide (PB/Tx/Az) plus 5% normal horse serum. For immunolabeling, free-floating sections were incubated in primary antibody overnight at 4°C. Sections were then rinsed in 0.1 M PB and incubated for 1 h at room temperature in a bridging secondary antiserum raised in donkey directed against goat IgG (diluted at 1:200 with PB/Tx; secondaries from Jackson ImmunoResearch Laboratories, Inc., West Grove, PA). The sections were subsequently rinsed in 0.1 M PB and incubated for 1 h at room temperature in goat peroxidase-antiperoxidase (PAP, diluted at 1:1000 with PB/Tx; goat PAP from Jackson ImmunoResearch Laboratories). The sections were then rinsed in 0.1 M PB (pH 7.2–7.4), and the labeling visualized using diaminobenzidine tetrahydrochloride (DAB) in a 0.2 M sodium cacodylate buffer (pH 7.2–7.4). The sections were subsequently rinsed, mounted on gelatin-coated slides, air-dried, dehydrated and coverslipped with Permount® (Fisher Scientific, Pittsburgh, PA). The sections were examined with an Olympus BHS microscope with standard transmitted light or Differential Interference Contrast optics. Several cases were mapped and one illustrative case among these with the most extensive labeling (RF73) is presented here first (Figures 1–**3**) using schematics adapted from the atlas of Paxinos and Watson (2009). We will refer to these schematics as we present the other cases as well, to indicate the location of PRV-labeled populations of neurons in those cases. The results for all cases are presented in tabular form as well (Table 1). Images of sections with PRV+ staining as detected with DAB were captured on a Nikon 90i microscope with a 10x or a 20x objective. Digital images (2560 × 1920 pixels) were acquired with Nikon's NIS Elements software, and minimally processed in Adobe Photoshop for **Figures 4–6**. PRV+ cells within PVN and NTS were mapped onto detailed schematic drawings of these structures, to show the subnuclear location of the labeled neurons (**Figure 7**).

**Table 1:** **Summary of the 8 PRV cases used to map the distribution of neurons involved in the brain circuitry regulating choroidal blood flow via the SSN**.

**Animal**	**RF67**	**RF70**	**RF7**	**RF75**	**RF73**	**RF87**	**R11**	**R12**	**Summary**	**Summary**
Survival	72 h	74 h	52 h	142.6 h	142.4 h	73.5 h	72 h	72 h	PRV+ Cell	PRV+ Cell
Injected amount	2 μl	3 μl	1 μl	0.3 μl	0.2 μl	0.3 μl	0.5 μl	0.5 μl	Groups	Groups
Type of PRV injection case	Choroid only	Choroid only	Choroid only	Choroid only	Choroid only	Choroid mainly	Choroid mainly	Choroid mainly	Choroid circuit	Facial circuit
	**PRV+ neurons present**	**PRV+ neurons present**	**PRV+ neurons present**	**PRV+ neurons present**	**PRV+ neurons present**	**PRV+ neurons present**	**PRV+ neurons present**	**PRV+ neurons present**	**PRV+ neurons present**	**PRV+ neurons present**
**MOTOR AND PREGANGLIONIC CELL GROUPS**										
SSN	Some	Some	Many	Many	Many	Some	Many	Many	Yes	No
M3	None	None	None	None	None	None	None	None	No	No
EW	None	None	None	None	None	None	None	None	No	No
M4	None	None	None	None	None	None	None	None	No	No
M6	None	None	None	None	None	None	None	None	No	No
M7	None	None	None	None	None	Negligible	Negligible	Negligible	No	Yes
**TELENCEPHALON CELL GROUPS**										
Nu Accumbens	None	None	None	None	Bilateral	None	None	None	Yes	No
SI	None	None	Bilateral	None	Right	None	None	None	Yes	No
**DIENCEPHALON CELL GROUPS**										
MPO	None	None	None	None	Bilateral	None	None	None	Yes	No
LPO	None	None	Bilateral	None	Bilateral	Left	None	Left	Yes	No
PVN	None	None	Bilateral	Right	Bilateral	Bilateral	Rt>Lt	Rt>Lt	Yes	No
ZI	None	None	Bilateral	None	Bilateral	Bilateral	Bilateral	Bilateral	Yes	No
LH	None	None	Bilateral	Bilateral	Bilateral	Right	Bilateral	Bilateral	Yes	No
Arc	None	None	Bilateral	Left	Bilateral	None	None	None	Yes	No
**MESENCEPHALON CELL GROUPS**										
PAG	None	None	Bilateral	None	Bilateral	Bilateral	None	Bilateral	Yes	No
MRF	None	None	None	None	Bilateral	None	None	Bilateral	Yes	No
RaD	None	None	None	None	None	Right	None	Bilateral	No	Yes
RaMed	None	None	None	None	None	None	None	Bilateral	No	Yes
RRF	None	None	None	None	Bilateral	None	None	None	Yes	No
**METENCEPHALON CELL GROUPS**										
PBL	None	None	None	Right	Right	None	None	None	Yes	No
PBM	None	None	None	Right	Right	None	None	None	Yes	No
KF/A7	None	None	None	None	Right	Right	None	None	Yes	No
LoC	None	None	None	None	None	Bilateral	None	None	No	No
SubLoC	None	None	None	None	None	Right	None	None	No	Yes
PnO/Gi	None	None	None	None	Bilateral	Right	Bilateral	Bilateral	No	No
PnG/LDTg	None	None	None	Left	Right	None	None	None	Yes	No
Sp5	None	None	Left	Right	None	None	None	None	Yes	No
A5	None	None	Bilateral	Right	Left	Right	Bilateral	Right	Yes	No
Nu Prepositus	None	None	None	None	None	Left	None	None	No	Yes
RaM	None	None	None	None	Bilateral	Right	Bilateral	Bilateral	Yes	No
PPy	None	None	Right	None	Bilateral	Right	None	None	Yes	No
RaPa	None	None	Midline	None	None	None	None	None	Yes	No
GiA	None	None	None	None	Right	None	Bilateral	Bilateral	Yes	No
LPGi	None	None	Right	None	None	Bilateral	Bilateral	Bilateral	Yes	No
**MYELENCEPHALON CELL GROUPS**										
VeSp	None	None	None	None	None	Right	None	None	No	Yes
Sp5	None	None	Right	Bilateral	Bilateral	Right	Bilateral	Bilateral	Yes	No
RaOb	None	None	None	Midline	Midline	Midline	None	None	Yes	No
LPGi	None	None	Bilateral	Bilateral	Bilateral	Bilateral	Right	Bilateral	Yes	No
Gi	None	None	None	Bilateral	Bilateral	Bilateral	Bilateral	None	Yes	No
IRt	None	None	Bilateral	Bilateral	Bilateral	Bilateral	Bilateral	Bilateral	Yes	No
RVLM	None	Right	Bilateral	Bilateral	Bilateral	Right	Bilateral	Bilateral	Yes	No
CLVM	None	None	Bilateral	None	Bilateral	Bilateral	None	None	Yes	No
NTS	Right	None	Right	Right	Bilateral	Right	Bilateral	Bilateral	Yes	No
Area postrema	None	None	None	None	Bilateral	None	None	None	Yes	No

*The left most column indicates the information provided in each row, the next 8 columns present results for each of the PRV cases with injections limited or nearly limited to the right choroid, and the last two columns summarize the conclusions as to which of the PRV+ populations belong to the choroidal circuit (due to labeling from the right choroid) vs. the facial motor circuit (due to labeling from the right orbicularis oculi). Rows of the same color represent groupings of related information. The first three (yellow) rows provide identification of the cases, the survival time, the amount injected into right choroid, and summary of the extent to which the injection was limited to right choroid. The next 7 rows indicate whether PRV+ neurons were seen in cranial nerve preganglionic neurons innervating ganglia innervating the eye (SSN and EW) or motor neuron pools innervating extraocular or periorbital muscles (M3, M4, M6, and M7). The remaining rows show the brain cell groups containing PRV+ neurons, broken down by colors into the five major brain subdivisions. For each case and brain region, the table indicates if there were no PRV+ neurons (none), some only on the right side (right), some only on the left side (left), some on both sides (bilateral), or more on the right than left (Rt>Lt). Brain structures are identified by abbreviations as in the list of abbreviations*.

### Immunofluorescence and pathway tracing with BDA

**Figure 8:**
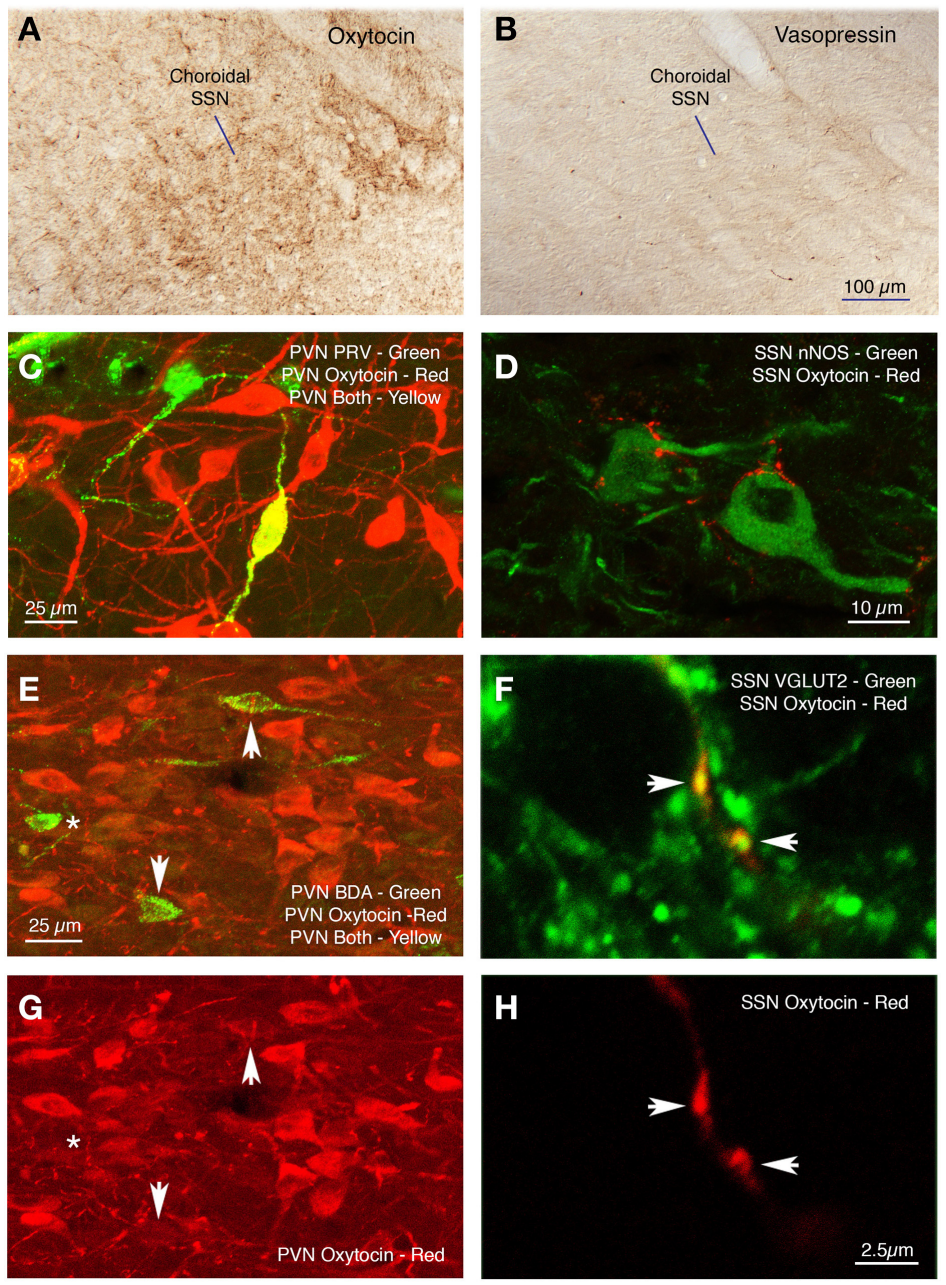
**Oxytocinergic terminals are abundant in prechoroidal SSN (A), but vasopressinergic terminals are rare (B)**. Immunofluorescence double labeling shows a PRV+ neuron (green) in dorsal parvicellular PVN that contains oxytocin (red) and thus appears yellow in the image **(C)**, and oxytocin-containing terminals ending on nNOS+ choroidal neurons of SSN **(D)**. **(E,G)** Show immunofluorescence double labeling for BDA (green) and oxytocin (red) in PVN, showing that two of the BDA+ cells are lightly labeled for oxytocin (arrows) and one is not (asterisk). **(F,H)** Show immunofluorescence double labeling for oxytocin (red) and VGLUT2 (green) in choroidal SSN. Note that both oxytocinergic terminals in SSN (arrows) are also positive for VGLUT2, as shown in the merged image for both **(F)**.

**Figure 9:**
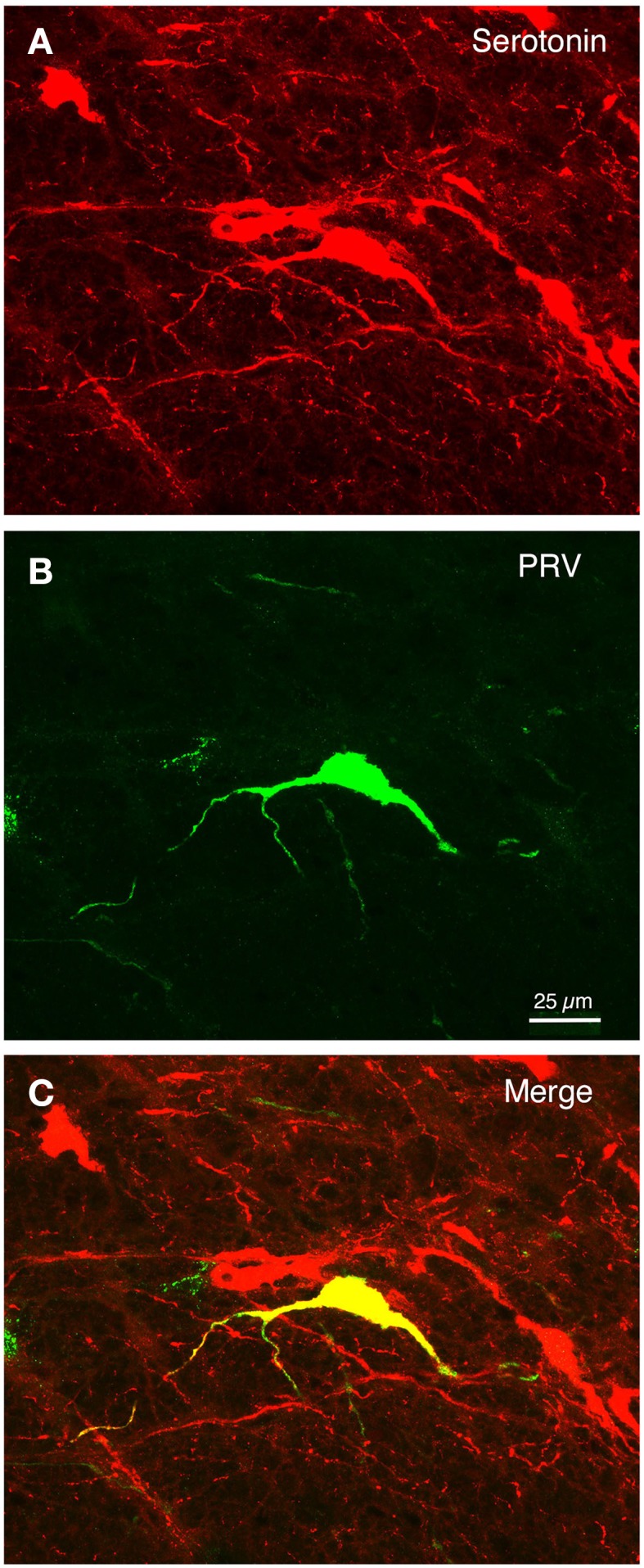
**The isolated PRV+ neurons of the B3 pontine midline region that were labeled by choroidal injection typically co-contained serotonin**. **(A–C)** Show a high power view of serotonergic neurons (red) in the RaM region **(A)**, one neuron of which was labeled with PRV (green) **(B)**, as shown in the merged image (yellow) **(C)**. Scale is the same in all images.

**Figure 10:**
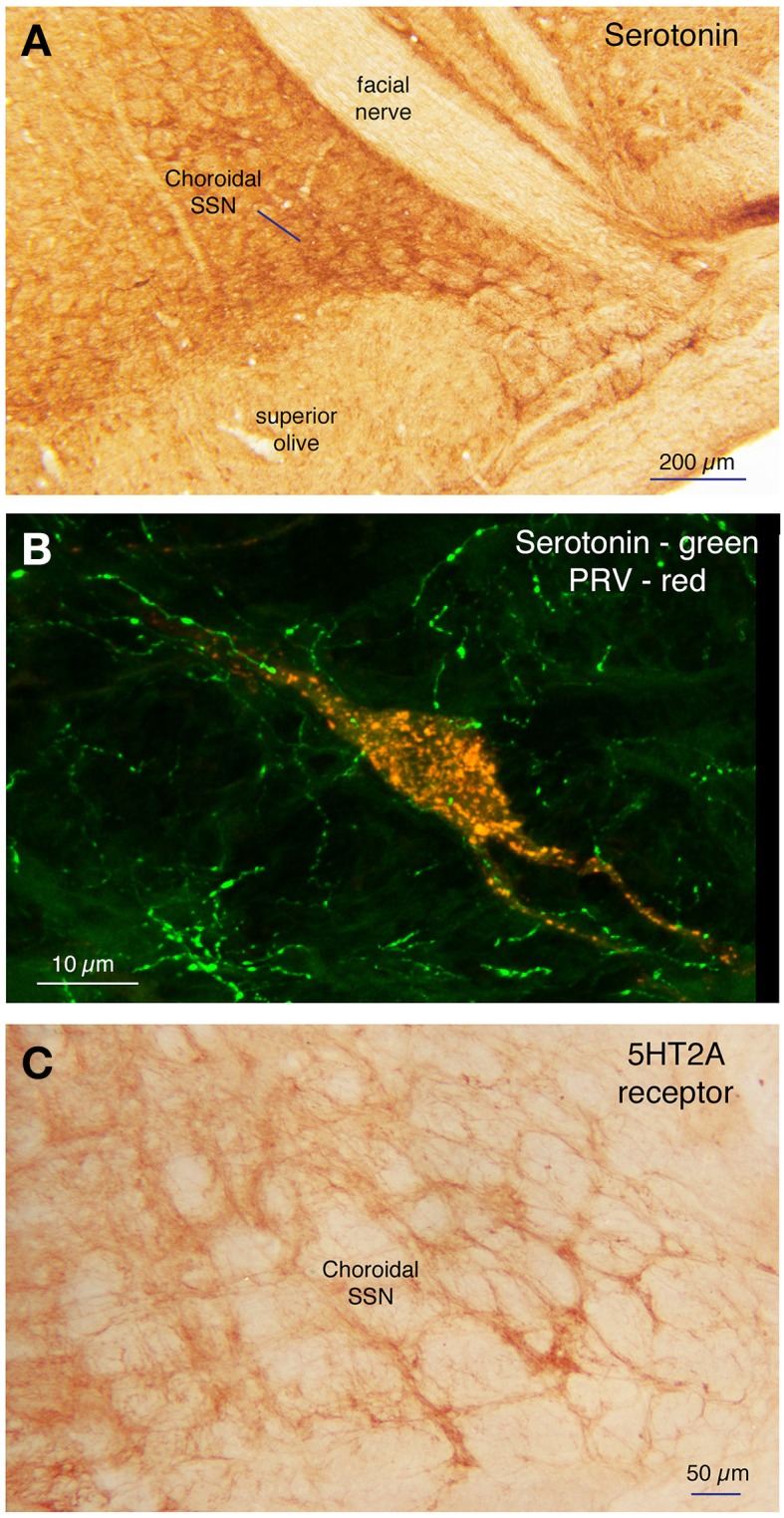
**Choroidal SSN is enriched in serotonergic terminals (A), and PRV+ SSN choroidal neurons (red) were seen to be contacted by serotonergic terminals (green) (B)**. Moreover, choroidal SSN is enriched in 5HT2A serotonin receptors **(C)**.

**Figure 11:**
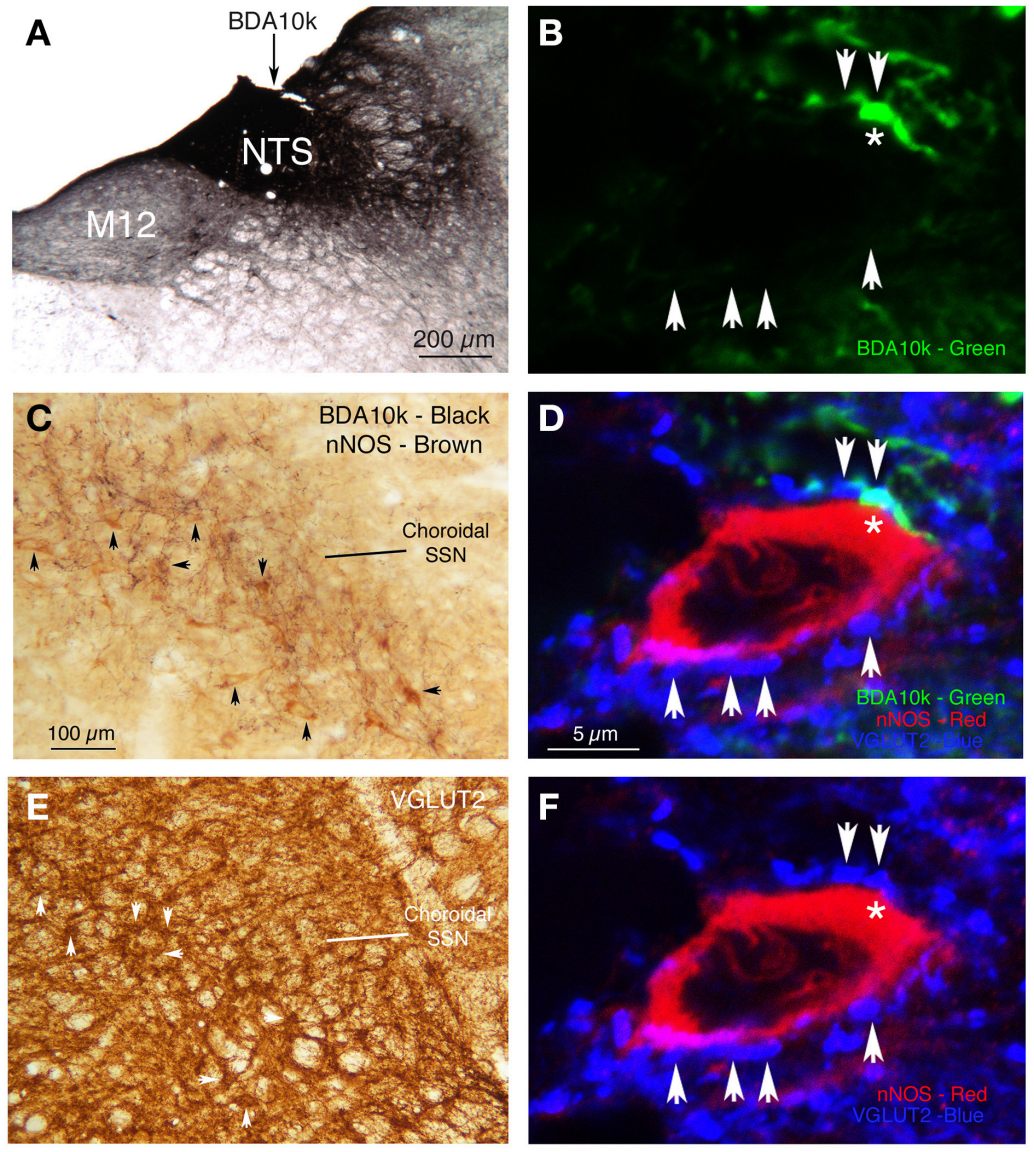
**(A)** BDA10k injection site in NTS that yielded the anterograde labeling in SSN shown in **(C)**. **(B)** The green channel from a section triple labeled by immunofluorescence, in this image showing BDA10k+ (green) terminal labeling from NTS. The red channel (showing nNOS labeling) and blue channel (showing VLUT2 labeling) are shown in **(D,F)**, respectively. Arrows indicate the location of blue VGLUT2+ terminals (shown in **F**) contacting the red nNOS+ perikaryon (shown in **D**). **(C)** Two color DAB double labeling showing brown nNOS+ prechoroidal neurons in SSN, overlapped by black BDA10k+ fibers arising from NTS. Some of the brown nNOS+ neurons are indicated by arrows. **(D)** Triple label immunofluorescence showing BDA10k+ (green) terminals from NTS, making VGLUT2+ contacts (blue) on NOS+ neurons (red) in SSN. Arrows indicate blue VGLUT2+ terminals contacting the red nNOS+ perikaryon shown. The asterisk indicates a green oxytocinergic terminal (as seen in the green channel alone in **B**) making contact with the red nNOS+ perikaryon. The image in **(F)** Shows that this terminal co-contains VGLUT2. **(E)** Brown DAB immunolabeling showing that VGLUT2 terminals are present throughout the SSN region, but more enriched in prechoroidal SSN. Note that clusters of VGLUT2+ terminals in some cases appear to outline perikarya (arrows). **(F)** Same image as in **(D)**, but only showing the many VGLUT2+ contacts (blue) on NOS+ neurons (red) in SSN. Scale in **(C,E)** is the same, and scale in **(B,D,F)** is the same.

**Figure 12:**
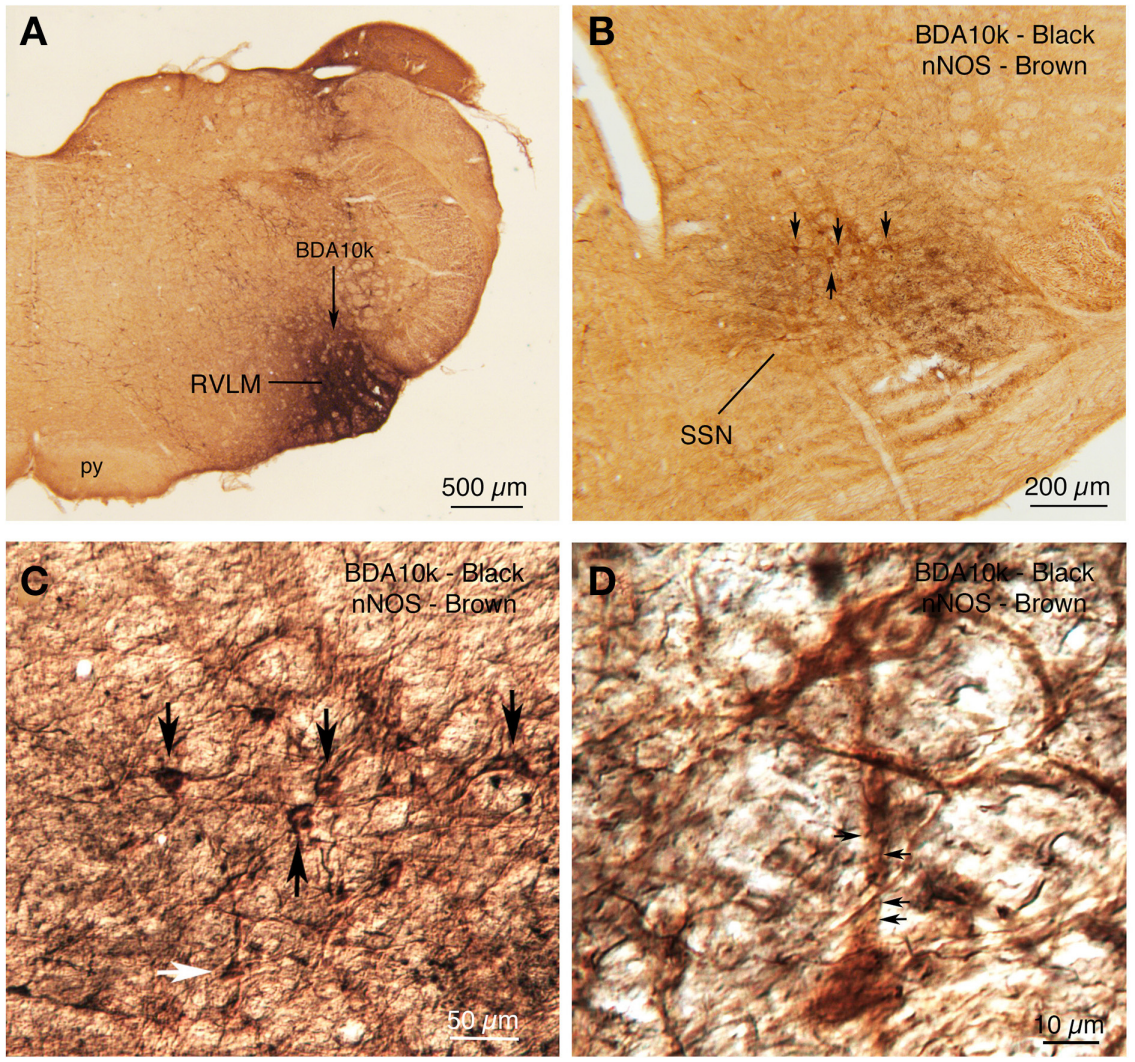
**(A)** BDA10k injection in RVLM that yielded the anterograde labeling in choroidal SSN shown in **(B)**. **(B)** Two color DAB double labeling showing brown nNOS+ prechoroidal neurons in SSN, overlapped by black BDA10k+ fibers arising from RVLM. Some brown nNOS+ neurons are indicated by arrows. **(C)** A differential contrast image (DIC) showing a higher power detail of the field shown in **(B)**. The same neurons indicated with arrows in **(B)** are indicated by arrows in **(C)**. One additional neuron is indicated by a white arrow and is shown at higher power in **(D)**. **(D)** The DIC image shows a brown nNOS neuron receiving contacts, some indicated by arrows, from black BDA10k+ fibers and terminals labeled from RVLM.

In additional studies, we characterized the neurochemistry of inputs to choroidal SSN from the major regions found to project to it, as revealed by PRV transneuronal retrograde labeling from choroid. In some of these studies, we used immunofluorescence to detect neurochemical markers in PRV+ neurons labeled from choroid or in neurons retrogradely labeled from choroidal SSN with biotinylated dextran amine 3000 kD (BDA3k). Among the PRV cases, we used two cases with injections confined to choroid presented here (RF73 and R11), and two cases not used here for the mapping of SSN circuitry because they did involve some slight spread to oculomotor or facial muscles. These cases are nonetheless used here for analysis of PVN and NTS neurons projecting to SSN because neither PVN nor NTS project to oculomotor or facial motor neuron pools (Hosoya et al., 1984, 1990; Geerling et al., 2010). We also used immunofluorescence double-labeling to confirm the neurochemical identity of specific sources of input to choroidal SSN, either by labeling the input to SSN with biotinylated dextran amine 10,000 kD (BDA10k) from its origin or by labeling it for its apparent neuropeptide content. We used this approach to confirm direct projections to SSN because vagaries in the timing of the trans-synaptic transport and amplification of PRV do not allow the between-case difference in the temporal order in which labeling occurs in specific regions to be a reliable guide for distinguishing sources of direct vs. higher order input to SSN (Card, 1998). Thus, for some regions labeled with PRV, we used light microscopic visualization of anterogradely transported BDA10k with a black nickel-intensified DAB reaction product to confirm specific inputs to choroidal SSN neurons, as identified with a brown DAB reaction product. Choroidal SSN neurons were visualized either by transneuronal retrograde labeling or by immunolabeling for nNOS. Anterograde and retrograde labeling with BDA was carried out as described previously, as was immunofluorescence and two-color DAB (Reiner et al., 2000; Li et al., 2010). Antibodies against the following substances were used: nNOS (Santa Cruz SC-648, raised in rabbit, used at 1:200–1:800), calbindin (Sigma C9848, raised in mouse, used at 1:5000), or tyrosine hydroxylase (Immunostar, raised in mouse, used at 1:2000), the type-2 vesicular glutamate transporter (VGLUT2, Sigma V2514, raised in rabbit, used at 1:1000), serotonin (Immunostar, raised in rabbit, used at 1:10,000), the 5HT2A serotonin receptor (Immunostar, raised in rabbit, used at 1:1000), oxytocin (provided by Harold Gainer, NIH, raised in mouse, used at 1:500), and vasopressin (provided by Alan Robinson, retired, raised in rabbit, used at 1:20,000). Sections labeled by immunofluorescence were viewed and images were captured using either a Nikon C1 or a Zeiss 710 confocal laser-scanning microscope. Confocal images were minimally processed in Adobe Photoshop for **Figures 8–11**. Light microscopic images of BDA or immunolabeling for **Figures 8, 10–12** were captured and processed as described above.

## Results

### Overview

Retrograde transport after tracer uptake from vitreous, extraocular muscles or periorbital facial musculature, and retrograde transport to brain was seen after our control injections into the vitreous or into the periorbital space. Such spread was evidenced by retrograde labeling in the nucleus of Edinger-Westphal (EW) in the case of vitreal spread, and by retrograde labeling in the oculomotor (M3) and/or trochlear nuclear complex (M4), or by retrograde labeling in the facial motor nucleus (M7) in the case of extrascleral spread. Note that the cytoarchitectonically defined EW in rats contains overlapping populations of centrally projecting urocortin-containing neurons and preganglionic neurons projecting to the ciliary ganglion (Kozicz et al., 2011). Only the latter are labeled by transneuronal transport due to virus injection into vitreous (which spreads to the pupil and ciliary body musculature to which the ciliary ganglion projects), and labeled neurons in EW after intravitreal PRV injection are thus preganglionic to the ciliary ganglion (Pickard et al., 2002). Labeling in the facial motor neuron pool was consistent with the expected location of motor neurons innervating the orbicularis oculi (Faulkner et al., 1997; Morcuende et al., 2002; Kurup et al., 2007), and labeling in the oculomotor neuron pools was consistent with the expected location of motor neurons innervating superior rectus and superior oblique muscles (Glicksman, 1980; Labandeira Garcia et al., 1983), respectively. From the cases in which we targeted choroid, we obtained eight with injections entirely or nearly entirely confined to choroid, as judged by the absence or near absence of retrograde labeling in EW, the extraocular motor neuron pools, or the facial somatomotor neuron pools, that also yielded labeling of SSN, as well as higher order labeling beyond SSN (Table 1). In general, specific higher-order labeling was obtained from choroid after either intermediate survival times (50–100 h) and larger injections, or long survival times (≥100 h) and smaller injections. The long survival times with smaller injections reflect the time needed for virus amplification after the minute injection of PRV into the choroid. Even for a similar size injection and survival time, however, there was variability in the extent of higher-order labeling. This variation may reflect the relative proximity of a given injection to intrachoroidal PPG fibers, which are not uniformly distributed in choroid (Stone, 1986; Stone et al., 1987; Reiner et al., 2010). In the following description of the distribution of labeled neurons after intrachoroidal PRV injection, we begin with the case with the most extensive labeling (RF73) to provide an overview of the central neurons that are part of the brain circuitry for controlling choroidal blood flow. We then present the other 7 cases, which aid in assessing which cell groups might project directly to choroidal SSN.

### Illustrative case with most extensive labeling beyond SSN following restricted injection of choroid (RF73)

Rat RF73 survived 143 h after a small injection (0.2 μl), but considerable higher-order labeling was seen, presumably due to an enrichment of PPG innervation at the choroidal injection site. Transneuronal retrograde labeling was substantial in choroidal SSN, but none was present in EW, the extraocular motor neuron pools or the facial motor nucleus (Table 1; Figures 1–2). Well-labeled neurons were observed as well throughout the brain (Figures 1–3). For example, moving in rostral to caudal order, a few neurons were seen ipsilaterally in the substantia innominata (SI) (Figures 1A–C), and bilaterally in the nucleus accumbens core (not shown), at the telencephalic level. Within the subthalamus and hypothalamus, labeled neurons were highly abundant bilaterally in the zona incerta (ZI) and paraventricular nucleus (PVN), respectively, but with an ipsilateral predominance (Figures 1C–F, 2A,C). Scattered labeled neurons were also seen bilaterally in the lateral (LPO) and medial preoptic areas (MPO), and the dorsomedial (DM), lateral (LH), posterior (PH), and arcuate (Arc) hypothalamus (Figures 1A–H, 2A, 4E,F). Within the midbrain, labeled neurons were seen in the periaqueductal gray (PAG) (Figures 2B–F, 5A,B), as well as in the mesencephalic reticular formation (MRF) and the retrorubral field (RRF) (Figures 2C–E). More caudally at isthmic levels, labeled neurons were seen in the ipsilateral lateral parabrachial region (PBL), the ipsilateral medial parabrachial region (PBM), the Kolliker-Fuse nucleus (KF) (bilaterally), the lateral dorsal tegmental nucleus (LDTg), and the isthmic reticular formation (bilaterally) (Figures 2F–H, 5C,D). Within the pons, well-labeled neurons were also seen in the raphe magnus nucleus (RaM), the alpha part of gigantocellular reticular nucleus (GiA), the parapyramidal nucleus (PPy), and A5 (Figures 2H, 3A,B, 5E,F). In the medulla, PRV+ neurons were seen in the nucleus of the solitary tract (NTS), the rostral ventrolateral medulla (RVLM), the caudal ventrolateral medulla (CVLM, which encompasses the A1/C1 region), the intermediate reticular nucleus (IRt), the lateral paragigantocellular nucleus (LPGi), the gigantocellular reticular nucleus (Gi), and the caudal spinal trigeminal nucleus (Sp5) (Figures 3C–F). Isolated labeled neurons were also seen in the area postrema (AP), and the raphe obscurus (RaOb) (Figures 3C–F). As considered in the Discussion, the cell groups labeled in RF73, but not in the following cases with less extensive labeling after selective choroidal injection are likely to project weakly to choroidal SSN, or indirectly via cell groups that project directly to SSN.

### Cases with labeling of SSN neurons and only slight higher-order labeling beyond SSN following restricted injection of choroid (RF67, RF70)

Two cases yielded labeling of a cluster of neurons in ventromedial SSN, but not in the extraocular muscle or facial muscle motor neuron pools or EW (Table 1), as well as higher-order labeling in either of two additional cell groups. In one case with a 72 h survival (RF67), a few labeled neurons were seen in the right lateral NTS just anterior to the obex (Figures 3D,E), as well as the aforementioned PRV+ neurons in the rostral ventromedial SSN. In a case with a 74 h survival (RF70), a few labeled neurons were seen in the rostral ventrolateral medulla (RVLM) (Figures 3C–E), in addition to the many in the right rostral ventromedial SSN (Figure 6A). The limited higher-order labeling in these two cases suggests NTS and RVLM to be major sources of input to choroidal SSN, as further evidenced in the subsequently discussed cases, and directly confirmed by the conventional pathway studies presented here.

### Cases with more extensive higher-order labeling beyond SSN following restricted injection of choroid (RF7, RF75)

A 52 h case (RF7) with PRV labeling of the choroidal control neurons of SSN (Figure 6B), but no labeling in the facial motor nucleus, EW or any oculomotor pool, showed higher-order labeling of neurons in several cell groups beyond the SSN (Table 1). For example, scattered PRV+ neurons were observed bilaterally in the lateral hypothalamus (LH), the arcuate hypothalamus (Arc), the lateral preoptic area (LPO), and the substantia innominata (Figures 1A–H, 4A, 6B). In the diencephalon, PRV+ neurons were observed bilaterally in the paraventricular nucleus (PVN), especially its caudal parvocellular part (Armstrong et al., 1980; Swanson and Kuypers, 1980), and bilaterally in the zona incerta (ZI) (Figures 1C–F). Labeled neurons in the midbrain were found only in the periaqueductal gray (PAG) (Figures 2B–E). Within the pons, in addition to the PRV+ neurons in SSN, labeled neurons were observed in the raphe pallidus (RaPa), the right LPGi, and the right parapyramidal nucleus (PPy) (Figures 2H, 3A,B). PRV+ neurons were also observed bilaterally in the A5 cell group, and in the left spinal nucleus of the trigeminus (Sp5) (Figures 2G,H). Rare labeled neurons were also observed bilaterally but with ipsilateral predominance in several medullary cell groups in rat RF7, notably the RVLM (Figure 6C), the CVLM, the lateral paragigantocellular nucleus (LPGi), and the region dorsal to the RVLM termed the intermediate reticular nucleus (IRt) (Figures 3C–E). Labeled neurons were also seen in the right caudal spinal trigeminal nucleus (Sp5) (Figure 3F). A few labeled neurons were observed in ipsilateral NTS, in its caudal and lateral part.

In rat RF75, the small (0.3 μl) intrachoroidal injection yielded labeling in choroidal SSN, but none in EW, or the extraocular or facial motor neuron pools following a 143 h survival time (Table 1). Anteriorly, we saw a few labeled neurons in the lateral hypothalamus, the arcuate region, and the right PVN, but none in the midbrain (Figures 1, 2). At the isthmic level we observed a few labeled neurons in the medial and lateral parabrachial nuclei (PBM and PBL, respectively) (Figure 2G), while in the pons we saw PRV+ neurons in the ipsilateral A5, the ipsilateral Sp5, and in the pontine gray (Figures 2G,H, 3A,B). We additionally observed transneuronal retrograde labeling bilaterally (with an ipsilateral predominance) in a few perikarya in the RVLM, the IRt, the lateral paragigantocellular nucleus (LPGi), the raphe obscurus (RaOb), gigantocellular reticular nucleus (Gi), and the caudal Sp5 of the medulla (Figure 3). Numerous labeled neurons were observed in ipsilateral, caudal lateral NTS (Figure 6D). Note that although the injection amount and survival time were similar in RF75 and RF73, more PRV+ neurons were observed in R73, presumably because of a greater enrichment of PPG innervation at the choroidal injection site in RF73.

### Higher-order intrachoroidal cases with higher-order labeling and slight spread to orbicularis oculi (RF87, R11, R12)

RF87 had prominent labeling of choroidal SSN neurons ipsilateral to the injection site, as well as of a few neurons in the facial motor nucleus (no more than one at any given level) (Table 1). In this case, in which the injected amount was small (0.3 μl), a limited number of higher-order PRV+ neurons were observed in many of the same regions as after the above noted injections restricted to choroid in RF7, RF73, and RF75. These regions included the LHA, ZI, LPO, and PVN of the hypothalamus (Figure 1). PRV+ neurons were also common in the PAG of the midbrain (Figures 2B–F). PRV+ neurons were seen in the LPGi, the parapyramidal nucleus (PPy), and A5 of the pons, and NTS, RVLM (Figures 6E,F), CVLM, IRt (Figures 6E,F), LPGi, Gi, and the Sp5 of the medulla. Additionally, rare individual labeled neurons were seen in the isthmic reticular formation, the raphe dorsalis (RaD), the right locus coeruleus (LoC), the subcoeruleus (subLoC), the Kolliker-Fuse nucleus (KF), the raphe magnus (RaM), the gigantocellular reticular nucleus (Gi), nucleus prepositus of the pons (Pr), the raphe obscurus (RaOb), and spinal vestibular nucleus (VeSp) at caudal medullary levels (Figures 2, 3). As considered further in Discussion, the labeling in some of these additional cell groups (e.g., spinal vestibular nucleus, nucleus prepositus of the pons, and the right locus coeruleus) is likely to stem from the slight spread of injected tracer to the orbicularis oculi in RF87, which resulted in slight retrograde labeling of facial motor neurons.

R11 had prominent labeling of choroidal SSN neurons ipsilateral to the injection site, and yet fewer labeled neurons in the facial motor nucleus than in RF87, following a 0.5 μl intrachoroidal injection and a 72-h survival (Table 1). A few PRV+ neurons were present in regions in which the above cases with restricted choroidal injections had shown labeled neurons, including: (1) scattered bilaterally, but generally with an ipsilateral preponderance, in the lateral hypothalamic area, ZI and PVN (Figure 4D); (2) bilaterally in A5, RaM, GiA, LDTg, and the lateral paragigantocellular nucleus (LPGi) in the pons; and (3) bilaterally, but generally with ipsilateral predominance, in NTS, RVLM, IRt, Gi, LPGi, and the caudal Sp5 of the medulla. Similar results were obtained in R12, whose injection and survival time were the same as R11, but which had slightly more facial motor nucleus labeling (Table 1). Differences included an absence of labeled neurons in Gi in R12, and the presence of a few labeled neurons in the isthmic and mesencephalic reticular formations, PAG, RaD, and the median raphe (RaMed) in R12.

### Analysis of PVN neurons projecting to choroidal SSN

Sections from RF73 and RF87 were mapped to characterize the location of neurons in PVN regulating the choroid via SSN. We found that PRV-immunolabeled neurons in PVN were especially abundant in its dorsal parvocellular subdivision (Figures 7A–D) (Armstrong et al., 1980; Swanson and Kuypers, 1980). Because both the oxytocinergic and the vasopressinergic neurons of parvocellular PVN are known to give rise to descending projections to hindbrain (Stocker et al., 2006; Yang et al., 2009), we immunolabeled sections through SSN from normal rat brain for oxytocin and vasopressin. We found that oxytocin-immunostained fibers are abundant in choroidal SSN, but vasopressin-immunostained fibers are nearly absent in choroidal SSN (Figures 8A,B, respectively). Consistent with this, oxytocin was observed in neurons in PVN that had been transneuronally retrogradely labeled from choroid with PRV or retrogradely labeled from SSN with BDA3k (Figure 8C), but vasopressin was not. In the case of BDA3k retrograde labeling, about half of the neurons labeled from SSN contained oxytocin. The oxytocinergic fibers in SSN were observed to contact choroidal SSN neurons (as identified by nNOS immunolabeling) (Figure 8D), and co-contain the glutamatergic terminal marker VGLUT2. Of further interest, we found that oxytocinergic fibers were also abundant in RVLM, where vasopressinergic fibers were again scarcer.

### Analysis of RaM neurons projecting to choroidal SSN

Within the pons, isolated well-labeled neurons were also seen in RaM, GiA, and the parapyramidal nucleus (PPy) (Figures 2H, 3A,B, 5E,F), which are known to contain the serotonergic neurons of the B3 group (Steinbusch, 1981). Moreover, the PRV+ neurons in RaM, GiA, and PPy resembled the serotonergic neurons known to reside in this region (Figure 9A). Consequently, we immunolabeled sections through RaM, GiA, and PPy from RF73 and RF75 for serotonin. We found that the PRV+ neurons in this pontine midline region commonly co-contained serotonin (Figures 9A–C). We additionally found that the part of SSN containing choroidal neurons was enriched in serotonergic terminals (Figure 10A), and that PRV+ SSN choroidal neurons were contacted by serotonergic terminals (Figure 10B). Moreover, we also found choroidal SSN was enriched in 5HT2A serotonin receptors (Figure 10C).

### Analysis of NTS neurons projecting to choroidal SSN

Two series of sections from RF73 were mapped to determine the location of PRV+ neurons in NTS regulating choroid. We found that the majority of PRV+ neurons were localized to the dorsal, intermediate and solitary tract subdivisions of NTS (Figures 7E–H), defining subdivisions as in Zhang and Ashwell (2001). To assess the NTS neuron types projecting to choroidal SSN, we immunolabeled sections through NTS with higher-order PRV immunolabeling from choroid or retrograde BDA3k labeling from SSN for several neurochemical markers enriched in subsets of NTS neurons. We found that the NTS neurons projecting to SSN did not label for neuronal nitric oxide synthase, calbindin, or tyrosine hydroxylase. Because of the physiological evidence that the NTS input to SSN is excitatory (Agassandian et al., 2002, 2003), we examined whether the terminals of NTS neurons in choroidal SSN are enriched in either of the glutamatergic terminal markers VGLUT1 or VGLUT2. We found that biotinylated dextran amine (10k) injections of NTS (Figure 11A) anterogradely labeled terminals that selectively overlapped and made contact with choroidal SSN neurons, as detected by nNOS immunolabeling (Figure 11C), and that choroidal SSN was rich in VGLUT2, but not VGLUT1 terminals (Figure 11E). Double-labeling immunohistochemistry showed that choroidal SSN neurons were contacted by numerous VGLUT2+ terminals (Figure 11D), and triple-labeling showed that BDA10k+ NTS terminals (Figure 11B) that contacted choroidal SSN neurons (as identified by nNOS immunolabeling) (Figure 11D) contained VGLUT2 (Figure 11F).

### Analysis of RVLM neurons projecting to choroidal SSN

Retrograde labeling from SSN with BDA3k also yielded labeled neurons in RVLM, further confirming it as a source of input to SSN (Figures 3, 6E,F). Anterograde BDA10K labeling from RVLM (Figure 12A) confirmed that it projects to choroidal SSN, as detected by nNOS immunolabeling (Figure 12B). Some BDA+ terminals could be seen to contact nNOS+ neurons.

## Discussion

The SSN contains the preganglionic neurons projecting to two different cranial parasympathetic ganglia via two different peripheral branches of the facial nerve, the PPG, via the greater petrosal nerve, and the submandibular ganglion, via the corda tympani (Contreras et al., 1980; Nicholson and Severin, 1981; Spencer et al., 1990; Jansen et al., 1992; Ng et al., 1994; Tóth et al., 1999). Preganglionic neurons innervating the PPG are located more ventrally within the SSN than those innervating the submandibular ganglion (Contreras et al., 1980; Nicholson and Severin, 1981; Spencer et al., 1990; Jansen et al., 1992; Ng et al., 1994; Tóth et al., 1999), and they control diverse cranial structures, including the lacrimal gland, the Meibomian glands, the orbital conjunctiva, choroidal blood vessels, the cerebral vasculature, and the nasal and palatal mucosa (Ruskell, 1965, 1971a,b; Uddman et al., 1980b; Ten Tusscher et al., 1990; Nakai et al., 1993; Van Der Werf et al., 1996; Schrödl et al., 2006). Our prior studies and our current study indicate that PPG choroidal neurons are a subset of the SSN neurons that occupy the rostromedial part of the nucleus, based on comparison to the entire preganglionic population labeled by PRV injections into the PPG (Spencer et al., 1990; Cuthbertson et al., 2003; Li et al., 2010). Moreover, the choroidal SSN neurons tend to reside rostromedial to those observed after injection of PRV into the Meibomian glands (Ledoux et al., 2001) or the lacrimal gland (Tóth et al., 1999). The regions in which we saw higher-order PRV labeling from the choroid in brain are consistent with those observed by Spencer et al. (1990) after PRV injection into rat PPG. The selectivity of our labeling, however, provides insight into the circuitry and mechanisms specifically underlying parasympathetic control of choroidal blood flow (ChBF) via SSN. As discussed below in more detail, the cell groups in which we observed labeled neurons after injections of PRV restricted to choroid have previously been implicated in the parasympathetic control of other cranial autonomic functions, including blood flow in other cranial structures (Jansen et al., 1992; Haxhiu et al., 1993; Izumi and Karita, 1994; Agassandian et al., 2003; Ishii et al., 2007), and in the sympathetic control of the peripheral systemic circulation (Strack et al., 1989a,b; Kerman et al., 2003).

### Technical considerations

Our goal was to identify brain regions controlling choroid via the SSN. To this end, we injected the transneuronal tracer PRV into choroid in 40 rats in which we also completely removed both superior cervical ganglia, to prevent transport of virus via the sympathetic innervation of the choroid (Tóth et al., 1999; Ledoux et al., 2001; Rezek et al., 2008). Using criteria described in the Materials and Methods section, from these we identified 8 rats with PRV injections into choroid with no spread or negligible spread outside of choroid, and higher order labeling in brain beyond SSN. The presence of a few PRV+ neurons in the facial motor nucleus in three of the eight cases (RF87, R11, R12), in a location consistent with spread of PRV to the orbicularis oculi muscle, indicated there was some apparent slight spread outside of PRV outside of the choroid in these cases (Faulkner et al., 1997; Morcuende et al., 2002; Gong et al., 2003; Kurup et al., 2007).

A prior PRV study of central labeling at various time points after PRV injection into the orbicularis oculi in rats (Morcuende et al., 2002) allows us to determine which PRV+ cell groups stemmed from the slight spread outside of choroid to orbicularis oculi in these three rats. Among the brain areas containing PRV+ neurons that were unique to rats RF87, R11, and R12 (i.e., not found in any of our other 5 cases with injections confined to choroid), all were labeled after orbicularis oculi injection in Morcuende et al. (2002), and/or have been shown to project directly to rat facial nucleus by conventional retrograde tracing methods (Hattox et al., 2002). These include RaD, RaMed, nucleus subcoeruleus, nucleus prepositus, and VeSp (Table 1). Of further note, Morcuende et al. (2002) did not observe labeling in PVN, the arcuate hypothalamus, RaM, PPy, GiA, SSN, A5, NTS, or VLM with <84 h survival after orbicularis oculi PRV injection. By contrast, we observed labeling in these cell groups with <84 h survival even in the three cases with slight spread to orbicularis oculi after intrachoroidal PRV injection. Thus, in neither these three cases, nor in our 5 cases with restricted choroidal injections, could labeling in PVN, the arcuate hypothalamus, RaM, PPy, GiA, SSN, A5, NTS, or VLM have arisen via spread along the facial motor pathway.

In the 8 cases presented here to describe higher-order PRV+ labeling from choroid, we also did not observe retrograde labeling in the motor neuron pools that control the extraocular muscles or the levator palpebrae muscle, and in no case did we observed PRV+ in EW preganglionic neurons controlling lens accommodation or pupillary constriction. Moreover, we did not see PRV+ neurons in the brain cell groups known to project to these motor neuron or preganglionic neuron pools. For example, vertical and horizontal gaze and vergence control centers are known to be located in the interstitial nucleus of the medial longitudinal fasciculus (iMLF) and the supraoculomotor area, and the paramedian mesencephalic and pontine reticular formations, respectively (Henn and Cohen, 1976; Nakao et al., 1986; Waitzman et al., 2000), and have direct projections to motor neuron pools controlling extraocular muscles in rats, rabbits, cats and monkeys (Steiger and Büttner-Ennever, 1979; Nakao and Shiraishi, 1983; Kairada, 1986; Nakao et al., 1986; Ostrowska et al., 1991; Kokkoroyannis et al., 1996; Ugolini et al., 2006). We did not see PRV+ neurons in the iMLF, the supraoculomotor area, or the paramedian reticular formation. Moreover, we also did not observe PRV+ neurons in central cell groups associated with EW circuitry, such as SCN and the olivary pretectal nucleus (Pickard et al., 2002; Smeraski et al., 2004). Thus, the higher order labeling also indicated no involvement of extraocular or EW circuitry in the labeling from the choroid that we report here.

Note that the absence of labeled neurons in EW in the cases reported here with PRV injections confined or largely confined to choroid suggests that, unlike in birds (Reiner et al., 1983, 1991; Cuthbertson et al., 1996), the EW-ciliary ganglion circuit in rats does not exert a major direct influence on control of ChBF. As this circuit in birds receives input from the suprachiasmatic nucleus (SCN), which also was not labeled in our 8 choroidal cases, and is responsible for light-mediated control of ChBF in birds (Fitzgerald et al., 1996; Shih et al., 1997), the light-mediated regulation of ChBF reported in piglets, monkeys and humans does not appear to be mediated by either SCN or EW in mammals (Parver et al., 1982, 1983; Bill and Sperber, 1990; Stiris et al., 1991; Longo et al., 2000). In this regard, the PRV+ neurons in the ventrocaudal part of the spinal trigeminal nucleus are of interest. This region receives corneal input (Aicher et al., 2014) and responds to bright light (Okamoto et al., 2009). It may thus be that corneal response to bright light can drive ChBF increases via a projection from cornea-responsive trigeminal nucleus neurons to the choroidal SSN.

### Higher-order labeling—SSN circuitry

#### PVN

**Figure 13:**
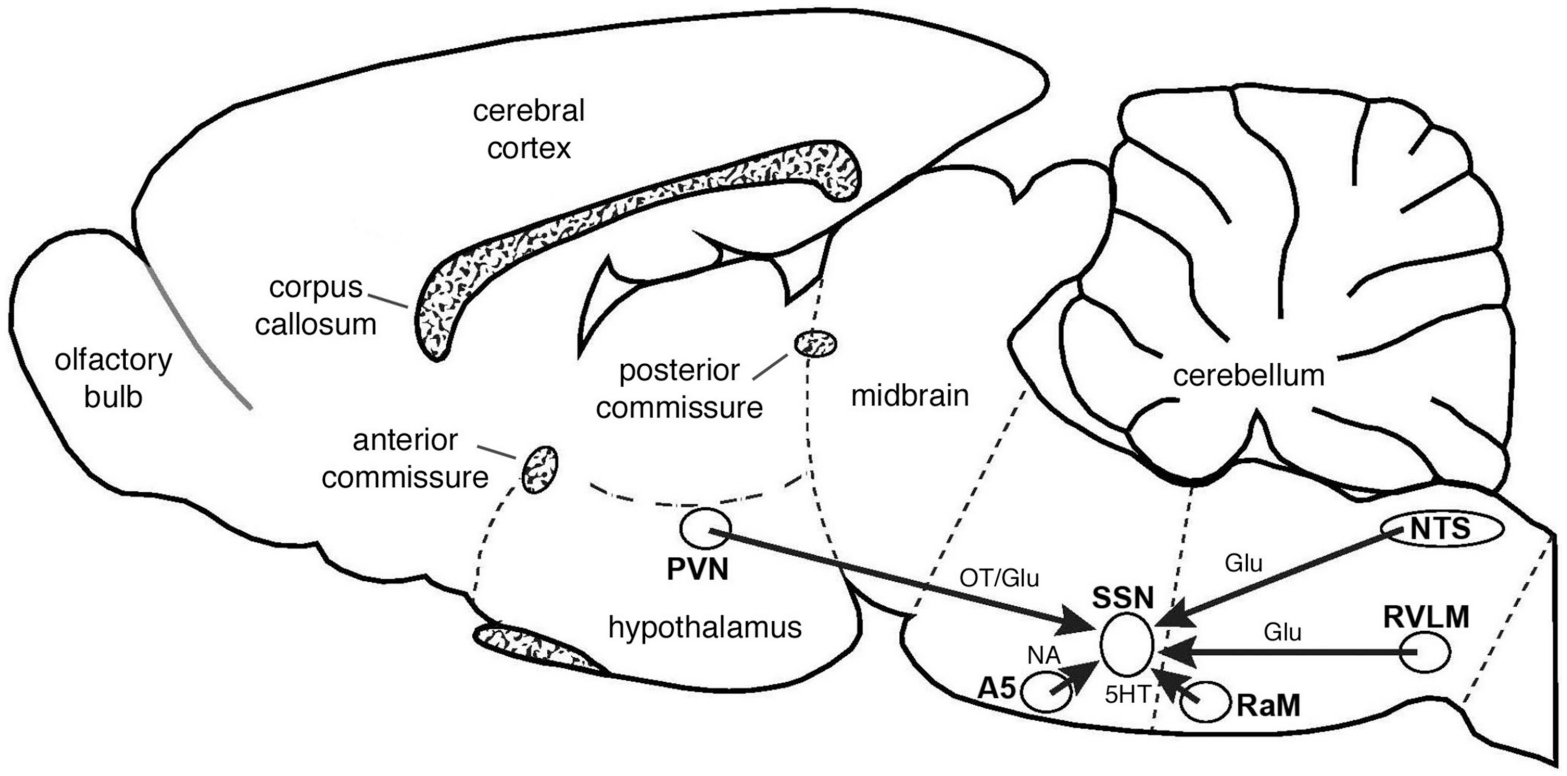
**Schematic summarizing the major inputs to SSN and their neurotransmitters, as interpreted from the present studies or from prior findings from the literature, as reviewed in the text**. Schematic drawing adapted from Paxinos and Watson (2009). Abbreviations: Glu, glutamate; NA, noradrenalin; OT, oxytocin; 5HT, serotonin.

Higher-order labeling was consistently observed in dorsal parvocellular PVN in cases with restricted or nearly restricted injections of PRV into the choroid. Consistent with this, PVN was found to contain higher-order labeling following PRV injection directly into the PPG by Spencer et al. (1990). Prior studies employing conventional pathway tracers have confirmed that PVN projects directly to SSN (Hosoya et al., 1984, 1990; Geerling et al., 2010), and recently we demonstrated by conventional pathway tracing methods that PVN input to SSN ends directly on choroidal neurons of SSN (Li et al., 2010). In the present study, we found that at least part of this projection arises from oxytocinergic PVN neurons, which end as glutamatergic oxytocin-containing terminals on nNOS+ choroidal SSN neurons (Figure 13). Consistent with this evidence for glutamatergic input to SSN choroidal neurons, SSN neurons have been found to be enriched in glutamate receptors and receive abundant synaptic input from excitatory terminals containing VGLUT2 (Kobayashi et al., 1997; Lin et al., 2003; Ishizuka et al., 2008). As diverse neuropeptides are found in PVN neurons with descending projections (Hallbeck et al., 2001; Lee et al., 2013), further studies will be needed to more fully characterize the neurochemical profile of PVN neurons projecting to choroidal SSN, but it seems clear that the descending PVN projections are especially enriched in oxytocin (Lee et al., 2013). Consistent with their direct projection to prechoroidal SSN, we have observed that activation of PVN increases ChBF in the ipsilateral eye (Fitzgerald et al., 2010). Given that the part of SSN that receives the PVN input controls both choroidal and cerebral blood flows (Geerling et al., 2010; Li et al., 2010), it may be that the PVN input influences both choroidal and cerebral blood flows. Our findings are of interest because parvocellular PVN is responsive to systemic blood pressure (BP) and blood volume, and it plays a role in maintaining stable systemic BP via projections to both sympathetic preganglionic neurons of the spinal cord and neurons of the RVLM that project to sympathetic preganglionic neurons of the spinal cord (Swanson and Kuypers, 1980; Sawchenko and Swanson, 1982; Porter and Brody, 1986; Wyss et al., 1994; Krukoff et al., 1997; Badoer and Merolli, 1998; Yang and Coote, 1998; Badoer, 2001; Godino et al., 2005; Guyenet, 2006; Stocker et al., 2006; Geerling et al., 2010). Oxytocinergic neurons of parvocellular PVN, but not vasopressinergic neurons, respond to hypotension and hypovolemia (Smith and Day, 2003) and mediate systemic sympathetic vasoconstriction and cardiac acceleration (Stocker et al., 2006; Yang et al., 2009; Nunn et al., 2011). The established role of PVN oxytocinergic neurons in systemic vascular responses to drops in systemic blood pressure or volume, via their outflow to sympathetic preganglionic neurons of the spinal cord, suggests that the oxytocinergic PVN input to SSN may contribute to the demonstrated adaptive regulation of ChBF in response to fluctuations in systemic blood pressure or volume (Kiel and Shepherd, 1992; Reiner et al., 2003, 2010, 2011). Thus, during an episode of diminished systemic blood pressure or volume, PVN may act to increase systemic blood pressure by sympathetic vasoconstriction in the periphery, via its output to sympathetic preganglionic neurons, and it may increase choroidal and cerebral blood flow by parasympathetic vasodilation within the eyes and brain by means of its projection to SSN.

#### NTS

We observed higher-order labeling in the dorsal, intermediate and solitary subdivisions of NTS in cases with restricted or nearly restricted PRV injections into choroid. The neurons of this part of NTS are highly enriched in VGLUT2 (Ziegler et al., 2012). In a previous study, we demonstrated by conventional pathway tracing methods that NTS input to SSN ends directly on its choroidal neurons (Li et al., 2010), and in the present study we observed that NTS-arising terminals ending on SSN choroidal neurons contain VGLUT2 (Figure 13). Consistent with our findings from PRV injection into choroid, NTS was found to contain higher-order labeling following PRV injection directly into the PPG in Spencer et al. (1990). Prior studies employing conventional pathway tracers have shown that NTS projects to SSN as an excitatory input, but did not specify the SSN subregion (Agassandian et al., 2002). Consistent with its direct projection to prechoroidal SSN, we have observed that activation of the NTS increases ChBF in the ipsilateral eye (Fitzgerald et al., 2010). The NTS also exerts a vasodilatory influence on cerebral blood flow and masseter muscle blood flow, presumably via a projection to SSN neurons that innervate PPG neurons regulating cerebral and masseter blood vessels (Nakai and Ogino, 1984; Golanov and Reis, 2001; Agassandian et al., 2003; Ishii et al., 2010). The subdivisions of NTS shown by our present findings to project to prechoroidal neurons of SSN receive aortic baroreceptor input via the vagus and glossopharyngeal nerves, and respond to systemic blood pressure fluctuation (Ciriello, 1983; Housley et al., 1987; Altschuler et al., 1989; Rogers et al., 1993; Mayne et al., 1998). Of note, NTS is also known to project directly, and polysynaptically via the PBL, to the PVN, and may thus be a major source of the responsiveness of parvocellular PVN to cardiovascular signals (Saper and Loewy, 1980; Calarescu et al., 1984; Goldstein and Kopin, 1990; Herbert et al., 1990; Weiss and Hatton, 1990; Ito and Seki, 1998). The role of NTS in systemic vascular responses to fluctuations in systemic blood pressure or volume is mediated via its projection to sympathetic preganglionic neurons of the spinal cord. This suggests that direct NTS input to SSN may also contribute to the demonstrated adaptive regulation of ChBF in response to fluctuations in systemic blood pressure or volume (Kiel and Shepherd, 1992; Reiner et al., 2003, 2010, 2011).

#### RaM (serotonergic B3 cell group)

The B3 serotonergic cell group of the hindbrain (spanning RaM, RaPa, PPy, LPGi, and GiA) has been implicated in sympathetic control of the systemic vasculature and shown to directly innervate sympathetic preganglionic neurons of the spinal cord. We found that the PRV-labeled neurons of RaM, RaPa, GiA and parapyramidal nucleus (PPy) after intrachoroidal PRV injection tend to be serotonergic (Steinbusch, 1981). They appear to give rise to serotonergic terminals on choroidal SSN neurons that appear to be enriched in serotonin 5HT2A receptors, which mediate excitatory responses (Figure 13) (Barnes and Sharp, 1999). Serotonergic neurons of the hindbrain B3 group are also activated by systemic hypotension (Dean and Woyach, 2004), and respond to stimulation of the aortic depressor nerve (Gao and Mason, 2001). By means of excitatory input to sympathetic preganglionic neurons in the spinal cord and to the RVLM, B3 neurons act to increase postcranial systemic vascular tone (Dampney, 1994; Bago et al., 2002; Ootsuka and Blessing, 2005). Consistent with this, the B3 serotonergic neurons have been shown to be critical for the sympathetic vasoconstriction that restores vascular tone after hypotensive hemorrhage (Kung et al., 2010). The serotonergic neurons of the raphe region projecting directly to SSN may thus act to excite prechoroidal SSN neurons, and thereby contribute to choroidal vasodilation during systemic hypotension.

#### A5 adrenergic cell group

The adrenergic A5 group of the pons has been implicated in sympathetic control of the systemic vasculature, and shown to have direct excitatory input to sympathetic preganglionic neurons of the spinal cord (Huangfu et al., 1991; Dampney, 1994). The noradrenergic neurons of A5 are also activated by systemic hypotension (Horiuchi et al., 1999), and inhibited by hypertension or stimulation of the aortic depressor nerve (Huangfu et al., 1991). The A5 neurons thus appear to promote systemic sympathetic vasoconstriction in response to hypotension. Assuming that A5 input to prechoroidal SSN is also excitatory, this input would also increase choroidal vasodilation during systemic hypotension (Figure 13).

#### RVLM

Spencer et al. (1990) showed that RVLM projects to SSN, by higher order labeling after PRV injection into PPG. Our results based on intrachoroidal injection of PRV reveal that choroidal SSN neurons are among the targets of RVLM. Our complementary conventional pathway tracing confirmed this and showed that RVLM projects specifically to choroidal SSN (Figure 13). The finding that RVLM activation increases cerebral blood flow is also consistent with a projection from RVLM to SSN, and suggests this projection is excitatory (Golanov and Reis, 1994, 1996; Reis et al., 1994; Cetas et al., 2009). The RVLM directly innervates sympathetic preganglionic neurons of the spinal cord, and thereby is involved in sympathetic control of the systemic vasculature. The RVLM receives GABAergic input from neurons of the CVLM, and these CVLM neurons receive excitatory input from baroreceptive NTS (Dampney, 1994; Wyss et al., 1994; Pilowsky et al., 1995; Fan et al., 1996; Reiner et al., 2003; Schreihofer and Guyenet, 2003; Kumagai et al., 2012). Activation of baroreceptive NTS by heightened blood pressure signals from the aortic depressor and carotid sinus nerves thereby leads to diminished outflow from RVLM to sympathetic preganglionic neurons, and reduces systemic vasoconstriction, which relieves the hypertension, and is part of the systemic baroreflex (Wyss et al., 1994; Fan et al., 1996; Schreihofer and Guyenet, 2003; Kumagai et al., 2012). Conversely, diminished systemic blood pressure leads to diminished activation of GABAergic neurons of the CVLM by baroreceptive NTS, and increased drive from RVLM to sympathetic preganglionic neurons, causing peripheral vasoconstriction to correct the blood pressure drop. The oxytocinergic excitatory input to RVLM from PVN may provide an additional input that drives systemic vasoconstriction during hypotension (Stocker et al., 2006). The projection from the RVLM to choroidal SSN may help promote choroidal vasodilation during low systemic blood pressure, thereby preventing underperfusion of the choroid.

#### Additional regions

Higher-order labeling was observed after intrachoroidal PRV in additional forebrain, midbrain and hindbrain components of brain autonomic circuitry. These structures include the substantia innominata at telencephalic levels, the zona incerta and diverse hypothalamic nuclei at diencephalic levels (including the preoptic area, the lateral hypothalamus, and the arcuate region), the periaqueductal gray (PAG), the retrorubral field at midbrain levels, and the parabrachial nucleus and Kolliker-Fuse nuclei at isthmic levels. Spencer et al. (1990) also observed labeling in these structures after PRV injections into PPG. Prior studies have reported that some of these structures (zona incerta, preoptic hypothalamus, lateral hypothalamus, arcuate hypothalamus, and PAG) appear to project directly to SSN (Berk and Finkelstein, 1982; Hosoya et al., 1984; Nemoto et al., 1995; Zardetto-Smith and Johnson, 1995; Kobayashi et al., 1997), while others such as the substantia innominata, retrorubral field, the parabrachial nucleus, and the Kolliker-Fuse nucleus at isthmic levels may largely project indirectly to prechoroidal SSN (Saper and Loewy, 1980; Allen and Cechetto, 1992; Guo et al., 2002). These various structures have been implicated in cardiovascular and thermoregulatory control by the parasympathetic and sympathetic nervous systems (Strack et al., 1989a,b; Barman, 1990; Dampney, 1994; Wyss et al., 1994; Jansen et al., 1995; Blair et al., 2001; Buijs et al., 2003; Nakamura et al., 2004; Blair and Mickelsen, 2006; Asahina et al., 2007; Nakamura and Morrison, 2008). Of note with regard to ChBF control, the part of the parabrachial nuclear complex in which we observed PRV+ neurons (the dorsal PBL and the waist region of the PBM) is known to receive blood pressure-related input from NTS, be responsive to hypotension, and play a role in the peripheral vasoconstrictive response to systemic hypotension (Herbert et al., 1990; Dampney, 1994; Blair et al., 2001; Blair and Mickelsen, 2006). Thus, the parabrachial input to SSN (regardless of whether it is direct or multisynaptic) may also drive choroidal vasodilation during systemic hypotension. Finally, Spencer et al. (1990) observed labeled neurons after PRV injection into PPG in regions where we did not observed labeled neurons after PRV injection into choroid. These regions include the amygdala, bed nucleus of the stria terminalis, paraventricular thalamus, the nucleus of the posterior commissure, and nucleus ruber. It is uncertain whether these regions are not part of the brain circuitry controlling choroidal SSN, or whether their connectivity with this circuit is too weak to have been detected by our minute PRV injections into choroid.

#### Summary

By means of transneuronal labeling with PRV from the choroid supplemented by conventional pathway tracing methods and immunolabeling, we implicated numerous central cell groups in the control of choroidal blood flow. These cell groups notably include the paraventricular nucleus of the hypothalamus, the periaqueductal central gray, the raphe magnus and the B3 region of the pons, the A5 cell group, the nucleus of the solitary tract in the medulla, the rostral ventrolateral medulla, and the intermediate reticular nucleus of the medulla. Many of these cell groups having input to SSN are responsive to systemic blood pressure fluctuations and involved in systemic sympathetic blood pressure regulation. The links between the central systemic sympathetic and ocular parasympathetic circuitries reinforces the possibility that control of the two operates in parallel—with the systemic sympathetic control serving to maintain blood pressure in the face of episodic declines in blood pressure, and the parasympathetic control of ChBF serving to maintain high ChBF during bouts of low systemic blood pressure. Consistent with the protective role that such parasympathetic ocular vascular control might play during hypotension, severing PPG input to the cerebral vasculature intensifies the cerebral damage occurring with an ischemic event (Kano et al., 1991; Koketsu et al., 1992).

## Author contributions

All authors carried out the PRV studies. The conventional pathway tracing and immunolabeling was carried out by CL and AR. The manuscript was written mainly by AR, CL, and MECF. The research was planned by AR.

### Conflict of interest statement

The authors declare that the research was conducted in the absence of any commercial or financial relationships that could be construed as a potential conflict of interest.

## Abbrevation

A1/C1: A1 noradrenaline/C1 adrenaline cell groups
A5: A5 noradrenaline cells
Ac: anterior commissure
ACo: anterior cortical amygdaloid nucleus
AD: anterodorsal thalamic nucleus
AH: anterior hypothalamic area
AHi: anterolateral part of amygdalohippocampal area
Alv: alveus of hippocampus
Amb: ambiguus nucleus
AP: area postrema
APir: amygdalopiriform transition area
APT: anterior pretectal nucleus
Arc: arcuate nucleus
ATg: anterior tegmental nucleus
AV: anteroventral thalamic nucleus
BLA: anterior part of basolateral amygdaloid nucleus
BLP: posterior part of basolateral amygdala
BMA: anterior part of basomedial amygdaloid nucleus
BMP: posterior part of basomedial amygdala
BSTL: bed nucleus of stria terminalis
C: central subnucleus of NTS in Figure 7
CA1: field CA1 of hippocampus
CA2: field CA2 of hippocampus
CA3: field CA3 of hippocampus
Cc: corpus callosum
CeA: central amygdaloid nucleus
cIMLF: caudal interstitial nucleus of mlf
Cl: claustrum
CL: centrolateral thalamic nucleus
CM: central medial thalamic nucleus
Com: commissural subnucleus of NTS in Figure 7
Cp: cerebral peduncle
CPu: caudate putamen
Cu: cuneate nucleus
Cu: cuneate fasciculus
CxA: cortical amygdala
DCN: dorsal cochlear nucleus
Den: dorsal endopiriform nucleus
DG: dentate gyrus
DHA: dorsal hypothalamic area
Dk: nucleus of Darkschewitsch
DLG: dorsal lateral geniculate nucleus
DM: dorsomedial hypothalamic nucleus
DP: dorsal parvocellular subdivision of PVN in Figure 7
Dtg: dorsal tegmental bundle
DTg: dorsal tegmental nucleus
Ecu: external cuneate nucleus
En: endopiriform cortex
Ent: entorhinal cortex
EW: Edinger-Westphal nucleus
F: fornix
FA: fornical area of PVN in Figure 7
Fr: fasciculus retroflexus
G: gelatinous subnucleus of NTS in Figure 7
g7: genu of facial nerve
Gi: gigantocellular reticular nucleus
GiA: alpha part of gigantocellular reticular nucleus
GPe: globus pallidus, external segment
GPi: globus pallidus, internal segment
Gr: gracile nucleus
HbL: lateral habenular nucleus
HbM: medial habenular nucleus
HDB: nucleus of horizontal limb diagonal band
IAM: interanteromedial thalamic nucleus
Ic: internal capsule
IC: inferior colliculus
IGL: intergeniculate leaflet
IM: intermediate subnucleus of NTS in Figure 7
InC: interstitial nucleus of Cajal
InG: intermediate gray layer of superior colliculus
InWh: intermediate white layer of superior colliculus
IO: inferior olive
IP: interpeduncular nucleus
IRt: intermediate reticular nucleus
ISN: inferior salivatory nucleus
KF/A7: Kolliker-Fuse nucleus/A7 noradrenalin cells
La: lateral amygdaloid nucleus
LD: laterodorsal thalamic nucleus
LDTg: laterodorsal tegmental nucleus
Lfp: longitudinal fasciculus of pons
LH: lateral hypothalamic area
Ll: lateral lemniscus
LL: nucleus of lateral lemniscus
LM: lateral mammillary nucleus
LoC: locus coeruleus
LP: lateral parvocellular subdivision of PVN in Figure 7
LPT: lateral posterior thalamic nucleus
LPGi: lateral paragigantocellular nucleus
LP_L_: lateral part of the lateral parvocellular subdivision of PVN in Figure 7
LPO: lateral preoptic area
LSO: lateral superior olive
LVPO: lateroventral periolivary nucleus
M: medial subnucleus of NTS in Figure 7
M3: oculomotor nucleus
M4: trochlear nucleus
m5: motor root of trigeminal nerve
M5: motor trigeminal nucleus
M6: abducens motor nucleus
M7: facial motor nucleus
M10: dorsal vagal motor nucleus
M12: hypoglossal motor nucleus
MCPO: magnocellular preoptic nucleus
MD: mediodorsal thalamic nucleus
MeA: medial amygdaloid nucleus
MeAD: anterodorsal part of medial amygdaloid nucleus
MGN: medial geniculate nucleus
Ml: medial lemniscus
ML: lateral part of medial mammillary nucleus
Mlf: medial longitudinal fasciculus
MM: medial part of medial mammillary nucleus
Mp: mammillary peduncle
MPdd: dorsal part of the dorsal medial parvocellular subdivision of PVN in Figure 7
MPdv: ventral part of the dorsal medial parvocellular subdivision of PVN in Figure 7
MPO: medial preoptic area
MRF: mesencephalic reticular formation
Mt: mammillothalamic tract
n7: facial nerve
n8: vestibulocochlear nerve
NOT: nucleus of optic tract
NTS: nucleus of solitary tract
OPN: olivary pretectal nucleus
Opt: optic tract
Ox: optic chiasm
Pa6: para-abducens nucleus
PAG: periaqueductal gray
PBL: lateral part of parabrachial nucleus
PBM: ventral part of parabrachial nucleus
PC: paracentral thalamic nucleus
Pc: posterior commissure
Pd: predorsal bundle
PDTg: posterodorsal tegmental nucleus
PF: parafascicular thalamic nucleus
PH: posterior hypothalamic area
PIN: posterior intralaminar thalamic nucleus
Pir: piriform cortex
PM: posterior magnocellular division of PVN in Figure 7
PMCo: posteromedial cortical amygdaloid nucleus
PMn: paramedian reticular nucleus
PMv: ventral part of the posterior magnocellular division of PVN in Figure 7
Pn: pontine nuclei
PnC: caudal part of pontine reticular nucleus
PnG: pontine gray
PnO: oral part of pontine reticular nucleus
PnV: ventral part of pontine reticular nucleus
Po: posterior thalamic nucleus
POA: preoptic area
PoMn: posteromedian thalamic nucleus
PPT: posterior pretectal nucleus
PPy: parapyramidal nucleus
Pr: prepositus nucleus
Pr5: principal nucleus of the trigeminus
PrC: precommissural nucleus
PRF: prerubral field
PT: paratenial thalamic nucleus
PV: periventricular region of hypothalamus in Figure 7
PVA: anterior part of paraventricular thalamic nucleus
PVN: paraventricular nucleus of the hypothalamus
PVP: posterior part of paraventricular thalamic nucleus
PVT: paraventricular thalamic nucleus
Py: pyramid
Pyx: pyramidal decussation
RaCLi: caudal linear nucleus of raphe
RaD: dorsal raphe
RaM: raphe magnus nucleus
RAmb: retroambiguus nucleus
RaMed: median raphe
RaOb: raphe obscurus nucleus
RaPa: raphe pallidus nucleus
RaPMn: paramedian raphe nucleus
RaPn: pontine raphe nucleus
RaRLi: rostral linear nucleus of raphe
RCh: retrochiasmatic area
Re: reuniens thalamic nucleus
Rh: rhomboid thalamic nucleus
RI: rostral interstitial nucleus of medial longitudinal fasciculus
RRF: retrorubral field
Rs: rubrospinal tract
RtTg: reticulotegmental nucleus of pons
Ru: nucleus ruber
RVLM: rostral ventrolateral nucleus of medulla
s5: sensory root of trigeminal nerve
SCN: suprachiasmatic nucleus
Scp: superior cerebellar peduncle
SI: substantia innominate
Sm: stria medullaris of thalamus
SNc: substantia nigra pars compacta
SNL: substantia nigra pars lateralis
SNr: substantia nigra pars reticulate
Sol: solitary tract and subnucleus of NTS in Figure 7
SON: supraoptic nucleus
sp5: spinal trigeminal tract
Sp5: spinal trigeminal nucleus
Sp5O: oral part of spinal trigeminal nucleus
SPO: superior paraolivary nucleus
SSN: superior salivatory nucleus
St: stria terminalis
STN: subthalamic nucleus
Sub: submedius thalamic nucleus
SubLoC: nucleus subcoeruleus
SuG: superficial gray layer of superior colliculus
SuM: supramammillary nucleus
SuOM: supraoculomotor area
TRN: thalamic reticular nucleus
Ts: tectospinal tract
Tth: trigeminothalamic tract
Tz: trapezoid body
Unc: uncinate fasciculus
V: ventral subnucleus of NTS in Figure 7
VAT: ventral anterior thalamic nucleus
VCN: ventral cochlear nucleus
VeL: lateral vestibular nucleus
VeM: medial vestibular nucleus
VeS: superior vestibular nucleus
VeSp: spinal vestibular nucleus
VL: ventrolateral subnucleus of NTS in Figure 7
VLT: ventrolateral thalamic nucleus
VLG: ventral lateral geniculate nucleus
VMT: ventromedial thalamic nucleus
VMH: ventromedial hypothalamic nucleus
VMPO: ventromedial preoptic nucleus
VP: ventral pallidum
VPL: ventral posterolateral thalamic nucleus
VPM: ventral posteromedial thalamic nucleus
VPO: ventral periolivary nucleus
VPPC: parvicelluar part of ventral posterior thalamic nucleus
VTA: ventral tegmental area
Xscp: decussation of superior cerebellar peduncle
ZI: zona incerta.
